# Potential medical applicability of *N*-β-Ala and* N*-His dipeptidomimetics against breast cancer: short in vitro and in silico screening

**DOI:** 10.1038/s41598-026-50964-7

**Published:** 2026-05-16

**Authors:** Klaudia Chmielewska, Justyna Budka, Katarzyna Kozłowska-Tylingo, Iwona Inkielewicz-Stepniak, Krystyna Dzierzbicka

**Affiliations:** 1 Department of Organic Chemistry, Faculty of Chemistry, Gdansk University of Technology, G. Narutowicza 11/12, 80233 Gdansk, Poland; 2 Department of Pharmaceutical Pathophysiology, Medical University of Gdansk, Debinki 7, 80211 Gdansk, Poland; 3 Department of Pharmaceutical Technology and Biochemistry, Gdansk University of Technology, G. Narutowicza 11/12, 80233 Gdansk, Poland; 4 Laboratory of Environmental Chemometrics, Faculty of Chemistry, University of Gdansk, Wita Stwosza 63, 80-308 Gdansk, Poland

**Keywords:** Breast cancer, Peptide, Beta-alanine, Histidine, Peptidomimetics, Cancer, Computational biology and bioinformatics, Drug discovery, Medical research

## Abstract

Short peptides conjugated to drugs are found to effectively improve wellbeing and recovery of cancer patients. However, to combat poor stability and bioavailability, their polar groups can be protected to form mimetics. Therefore, here we present synthesis, short biological screening and detailed computational analysis of 18* N*-terminal β-alanine and L-histidine dipeptidomimetics, to estimate their preliminary relevance as cancer therapeutics intermediates for conjugation. While extended biological experiments are deeply relevant to define drug functionality, they also require extensive financial resources. Herein, we employed computational methods to better estimate possible activities of the compounds, and create a perspective between in silico and in vitro to hypothesize on the acquired results. Most efficient representatives of each group were β-Ala-Ala (BAA) and His(Bn)-Val (HV) dipeptidomimetics, which seem to show signs of selectivity. Our research provides detailed theoretical data for scientists searching for their new conjugative agents to study. We hope to encourage other experts to incorporate computational methods into their research, to increase clarity and available data for further research.

**Supplementary Information:**

The online version contains supplementary material available at 10.1038/s41598-026-50964-7.

## Introduction

Chemotherapy—though effective—can be a very exhausting process for patients due to a number of adverse effects caused by toxic drugs not being able to distinguish a healthy cell from the malignant one^1^. Target specificity is a very desirable trait although rare, given that cancer cells imitate healthy cell membrane receptor patterns^2^. Moreover, cancer cells incorporate amino acids to help facilitate their metabolic needs, just like healthy cells, although their preferences vary^3–7^. While alanine is a canonical amino acid endogenously required for homeostasis—it seems to be deleterious to cancer cells. However, a variation of this amino acid, beta-alanine, was found to be preferentially up-taken by malignant cells and used to support cancer progression^3,4^.

Peptides and amino acid were found to substantially impact cancer metabolism^8,9^, and are usually proposed as a supplementary treatment to help transport cancer drugs towards specific receptors^10–12^. A great example of an anti-cancer peptide is carnosine, (β-Ala-His), endogenous substance able to ameliorate spleen and kidney toxicity after cisplatin-based chemotherapy^13^, with anti-aging effect on both muscles and the brain^14–16^. Although short peptides are functional and active, their stability is very fragile. Carnosine is no exception, active for only 5 min upon oral distribution. However, many derivatives of carnosine were found to maintain similar medical functionality, while being more stable^16–19^.

Replacing several parts of the peptide or conjugating its core to another drug can potentially be an attractive way to improve both its stability and functionality^20^. In fact, drug-peptide conjugates were found to alleviate the discomfort of chemotherapy patients and improve their well-being by allowing the drugs to selectively target cancer cells^21–23^. However, peptides with stabilizing protective groups will now turn into peptidomimetics, due to the changed structural features. Such mimetics were found attractive against a variety of cancer types, diabetes or immune dysfunctions, as well as bacterial and viral diseases like COVID-19 or influenza^24–30^. Examples of such peptidomimetics are: RGD derivatives (Arg-Gly-Asp)^31–34^, gastrin peptidomimetics^35^ and bombesin-drug conjugates^36^.

Functionality of carnosine seems to stem from both beta-alanine, as well as histidine. Interestingly, incorporation of even one β-amino acid to a natural peptide was found to enhance stability of its conjugative partner by offering a higher resistance against proteolytic enzymes, proven with both in vitro and in vivo studies^37^. Moreover, inverse carnosine (His-β-Ala) was also found an attractive anti-cancer agent, with IC50 against HepG2 hepatocellular carcinoma cell line valued at 11.23µg/mL, whereas carnosine was found with 10.01 µg/mL, hence suggesting that both *N*-His and *N*-β-Ala could be attractive starting points to creating new medical substances like drugs or adjuvants^38^.

Unfortunately, histidine decreases the solubility of its compounds, which can be fought by introducing structural changes. *N*-benzoyl derivatives of amino acids were found to attenuate cisplatin-induced nephropathy, suggesting that protecting histidine’s imidazole with a benzyl group might help increase the solubility, without reducing the activity of the final dipeptidomimetic^39^. Arginine is another polar amino acid with high polarity and positive charge, just like histidine. Interestingly, nitro-L-arginine methyl ester (L-NAME), a derivative with guanidine group protected with a NO_2_ and carboxyl group blocked with CH_3_—was found to inhibit angiogenesis, and has been suggested as adjuvant for cancer drugs^40^. Moreover, L-NAME incorporated into short peptide chains was found to support peptide-drug conjugates in their anti-cancer activity, creating such derivatives as CEP1612 and 13F-1^41,42^. In fact, methyl esters of peptides are also abundant in cancer research and such protection of carboxyl groups generally seems to increase the functionality of the final drug^43–48^.

Peptidomimetics conjugated with drugs like florouracyl, cisplatin or doxorubicin—are very interesting approaches towards the increase of selectivity and functionality of existing cancer treatments. Nevertheless, peptidomimetics of interest should also be analyzed separately, to better map and understand the functionality they offer to the final conjugate. However, given the available abundance of amino acid combinations as well as significant expenses of biological experiments—the search for medically relevant peptidomimetics should be assisted with computational in silico methods, like molecular docking, inverse docking, enrichment analysis, and ADME (theoretical estimation of metabolism, stability, drug-likeness, gastrointestinal absorption and blood–brain barrier passage). Our study aims to highlight the benefits of including said experiments in the basic research for drug design. Such additional computational analysis can be even more useful for chemists with a limited budget, as they steer the focus of researchers towards more attractive candidates and only those end up being selected for synthesis.

In this research work, we explored the preliminary potential of protected 18 dipeptidomimetics with either β-Ala or His(Bn) present at their *N*-terminal position. Given their resemblance to endogenous peptides, we propose them to be potential conjugate partners for cancer drugs, with the purpose to create drug-peptidomimetic hybrids similar to those already published^41,43,44,46^.

We’ve considered 10 out of 20 proteinogenic amino acids. The literature suggests that polar charge of amino acids can enhance their anti-cancer effect, therefore we’ve decided to exclude amino acids with uncharged side chains (serine, threonine, asparagine, glutamine). Moreover, due to the specificity of Sulphur compounds—we’ve also decided not to consider methionine and cysteine. Furthermore, given that our study is a proposal, rather than an in detail analysis, we’ve only included one amino acid with aromatic side chain—phenylalanine (and therefore, excluded: tyrosine, tryptophan, and cyclic proline. Last, but not least, we didn’t consider lysine, as highly polar amino acids are generally much less soluble, and therefore struggle to be cytotoxic without the inclusion of other biologically active side chains. Therefore, we’ve decided to only consider 2 out of 3 amino acids with electrically charged side chains (arginine due to its confirmed anti-cancer functionality in methyl ester form; and histidine as a main amino acid component, next to beta-alanine).

While inclusion of all 20 proteinogenic amino acids would be more precise, the manuscript’s goal was to investigate overall potential of anti-cancer functionality of dipeptidomimetics with beta-alanine or histidine; rather than full investigation of each possible variation. We hope to inspire experienced cancer scientists to further justify and investigate their own variations of dipeptidomimetics.

Cytotoxicity of proposed dipeptidomimetics was measured against MCF7 and HDFa (cancer and healthy in vitro cell line, respectively) and then possible functionality was analyzed with computational methods, including docking and enrichment analysis. Such combined methodology allowed for more insightful screening and additional hypotheses to explain in vitro results.

Detailed biological evaluation of nearly 20 chemical substances is a very expensive project, and while it is crucial to establish medical relevance—it can be successfully assisted with theoretical in silico methods to steer our attention towards the most attractive candidates. Although activity can still change after conjugation, exploring possible properties and metabolic routes of sole peptidomimetics is significantly useful, serving as an additional layer for comparative analysis with a conjugate of choice, as well as a drug compatibility suggestion for other researchers.

## Results

### Synthesis

While all *C*-terminal amino acids had been protected through methyl esterification (with both carboxyl groups protected in case of aspartic and glutamic acid), each *N*-terminal was accompanied by hydrochloride in ionic form. Further, highly polar guanidinium group of arginine was protected via NO_2_, deeply reducing alkaline pH of this amino acid as well as its nucleophilicity—therefore changing its native mode of action, though earlier studies show that such protected derivative of arginine can be even more useful as an intermediate in cancer treatment^40,49,50^. Last but not least, histidine aromatic component, imidazole, was protected with a benzyl group to increase solubility of the final dipeptidomimetic. However, impairing the visibility of histidine through imidazole modification can result in different cytotoxicity of the final dipeptidomimetics, which is later slightly explored through imidazole deprotection of two most potent His derivatives and comparison of their cytotoxicity to the protected constituents.

**Fig. 1:**
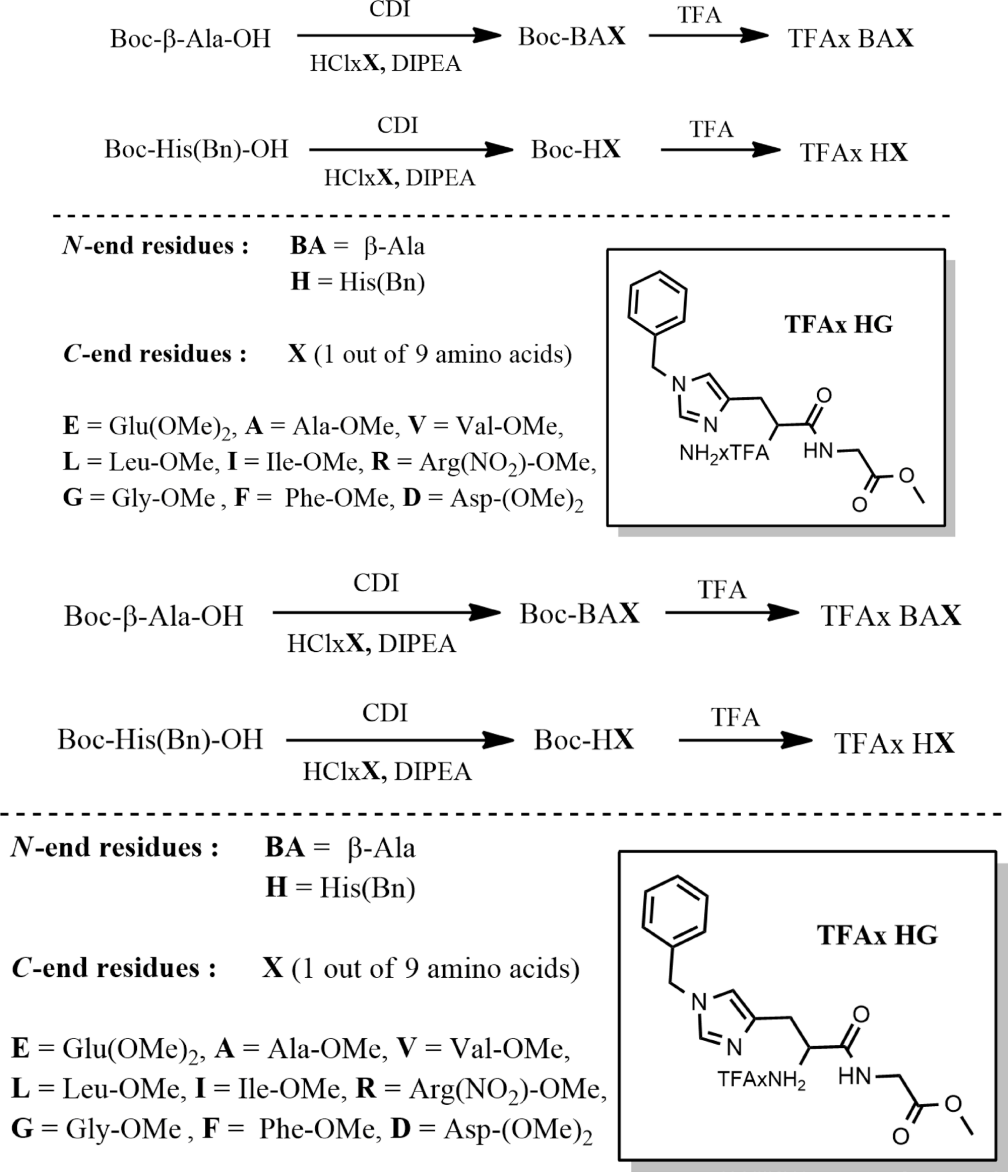
General synthesis procedure for dipeptidomimetics, shortcut explanation and structural formula of TFAx HG, shown as an example.

**Fig. 2:**
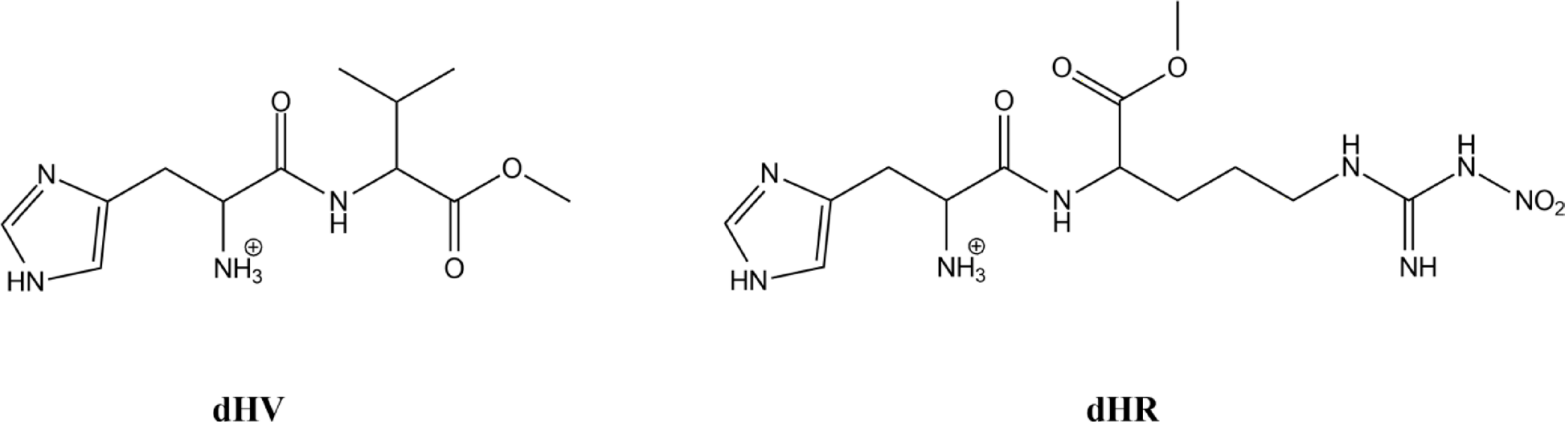
Structures of **dHV** and **dHR**, with deprotected imidazole rings.

All dipeptidomimetics were prepared using CDI-mediated one-pot peptide synthesis, a method where by-products are easily removed throughout the process^51–55^. Further, Boc-protective group was detached using trifluoroacetic acid (TFA) and dry products were used to create stocks for in vitro cytotoxicity assessment. Two protected histidine dipeptidomimetics (TFAx **HV** and TFAx **HR**) were identified as most cytotoxic histidine derivatives towards MCF7 breast cancer cell line. In order to determine whether the imidiazole protection is responsible for the anti-cancer effect, additional cytotoxicity test was performed on deprotected derivatives **dHV** and **dHR** (Figs. 1 and 2).

### Biological studies

Initial screening for potential cytotoxicity of 18 dipeptidomimetics with free *N*-terminal (TFAx dipeptidomimetics) was determined through MTT test, using MCF7 breast cancer cell line in a concentration of 10, 100, 500, 1000, 2500 and 5000 µM. Although in the context of cancer drugs, such amounts as 1mM and more—are too high to be considered pharmacologically relevant, such concentrations do not alleviate far from inhibitory concentration of natural peptides. Given that dipeptides are natural compounds, their derivatives are not expected to substantially disrupt human metabolism in small doses. Most cancer drugs are administered in a range within 10–100 µM, while research on amino acids and peptides focuses on much higher concentrations, usually in millimolar range, between 1 and 100 mM. Moreover, carnosine, dipeptide composed of beta-alanine and histidine, was found to affect MCF7 breast cancer cell line upon administration of 100 mM, which further validated our idea of the initial screening to be set on 1 mM, mirroring the examples found in the literature^56–60^. Finally, although effectivity in 1 mM concentration would suggest a moderate toxicity—it can still suggest potential as a supplement or conjugation partner.

Interestingly, activity of all dipeptidomimetics didn’t vary between 10 and 100 µM concentration, which suggests that those compounds are not effective against MCF7 cells within that dosage. Moreover, results for 2.5 and 5mM concentration of any tested compounds were found to be inconclusive compared to lower dosages.

**Fig. 3:**
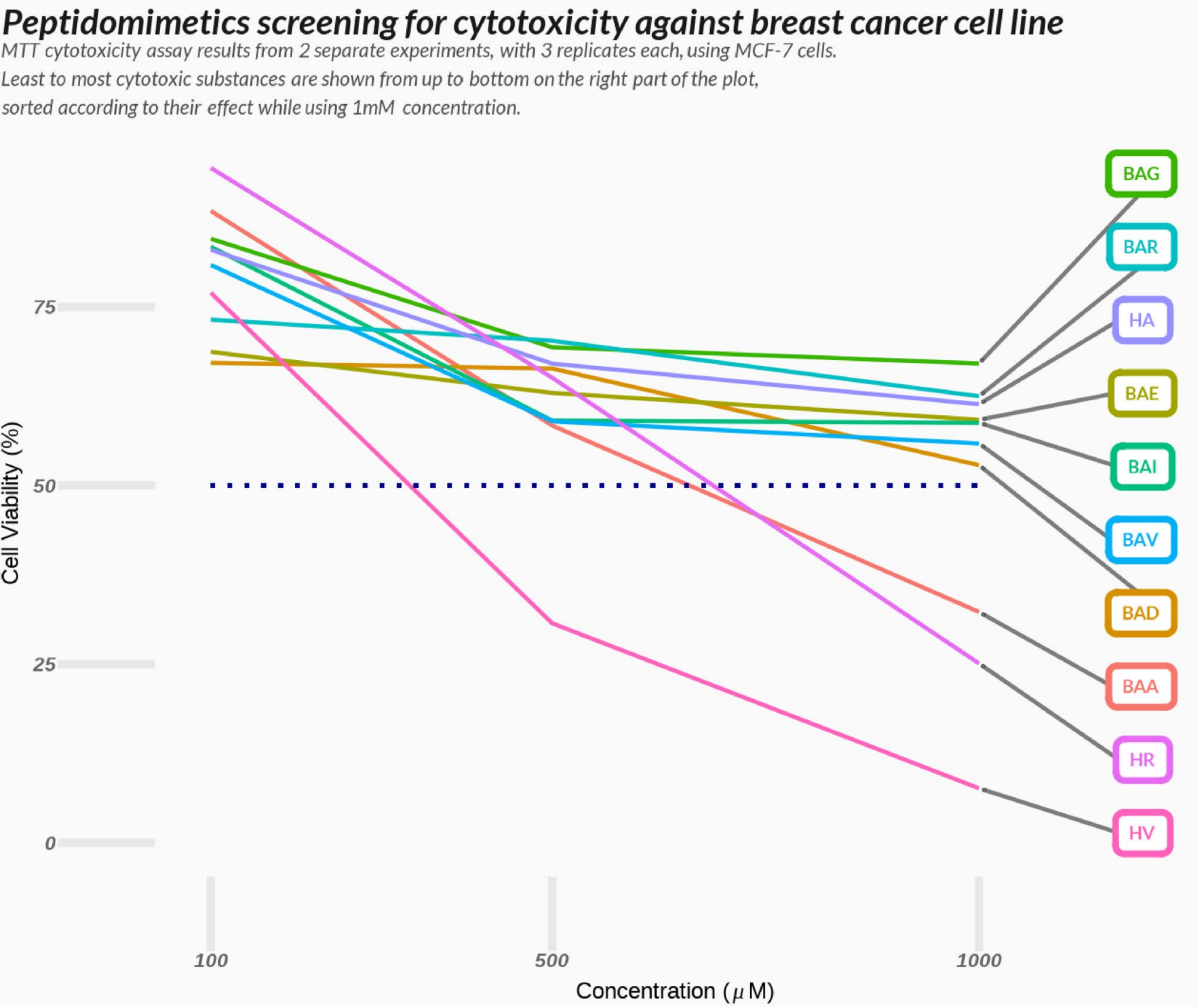
Cytotoxicity MTT in vitro test results, presenting 10 most potent substances of 18 tested. Mean ± SD was count based upon 2 independent experiments for each cell line; Y axis represents percentage of viable cells, in comparison to untreated control (DMSO-treated cells, where DMSO did not exceed 0.5%). Labels are sorted from least to most toxic, according to their effect on MCF7 viability in 1 mM concentration.

Interestingly, upon 1 mM, only 10 out of 18 proposed dipeptidomimetics decreased the viability of MCF7 cells by more than 25%. Moreover, only 3 of those 10 compounds were histidine derivatives. However, two of those, **HR** and **HV**—were also the most cytotoxic against the breast cancer cell line. Besides them, only **BAA** was able to kill more than 50% of viable cells. Those results are visually plotted in Fig. 3. Detailed results, together with data for concentration equal to 10, 2500 and 5000 µM, are presented in Supplementary Data.

Next MTT test was prepared for **BAA**, **HV**, **HR** (3 most cytotoxic compounds from the last test) and **HA** for comparison with **BAA**. Moreover, we’ve included carnosine as additional reference, given that its effect on MCF7 was recently reported in the literature (EC50 around 186mM and around 70–90% viability within concentration range of 1-100mM)^56^. Now the analysis was expanded to 3 independent experiments, MCF7 and HDFa cell lines, and concentration 100, 500, 1000 and 2500 µM (0.1, 0.5, 1.0 and 2.5 mM respectively).

HDFa is a non-cancerous cell line derived from fibroblasts, abundantly used by research groups focusing on beta-alanine and histidine derivatives^38,61,62^. Moreover, HDFa was recently established to be appropriate control towards breast cancer cells^63–66^, exhibiting same uptake pattern^63^, autophagy^67^ and cell cycle progression^68^. While the sole inclusion of 1 healthy cell line is not enough to draw solid conclusions, our goal was to estimate potential of dipeptidomimetics, rather than establishing and defining their true effectivity. Our study is exploratory and more studies would need to be performed in order to formulate solid conclusions.

**Fig. 4:**
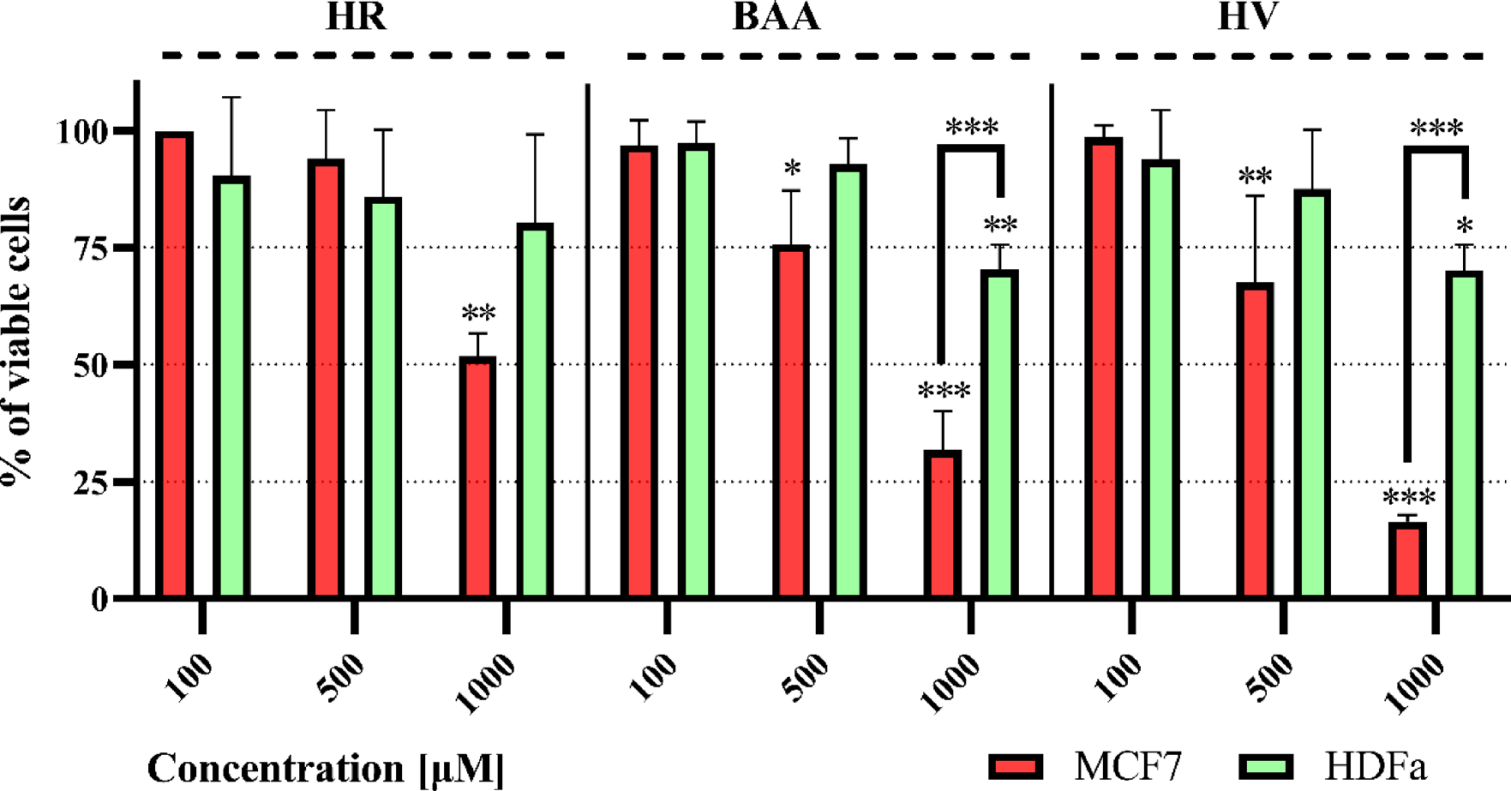
Detailed graph on ability to reduce the amount of viable cells by 3 most interesting compounds (TFAx **HR**, **BAA** and **HV**) against MCF7 (red bars) & HDFa (green bars) after 48 h of incubation. Mean ± SD was count based upon 3 independent experiments for each cell line; Tukey’s test: *p < 0.05; ***p < 0.001 Y axis represents percentage of viable cells, in comparison to untreated control (DMSO-treated cells, where DMSO did not exceed 0.5%).

Extended cytotoxicity experiments revealed that in 500 µM (0.5mM) concentration, TFAx dipeptidomimetics **BAA** and **HV**—were effective against MCF7 cancer cells; whereas **HR** was not. However, dipeptidomimetics did not seem to be selective, with both **BAA** and **HV** seemingly decreasing the viability of HDFa healthy line. Cytotoxicity for those 3 most effective dipeptidomimetics is visualized on Fig. 4. More detailed graph and raw viability counts, with additional data for TFAx HA/ HF/BAF can be found in Supplementary Data.

Concentration killing 50% of the MCF7 breast cancer cell culture (**IC**_**50**_) for TFAx **BAA**, **HV** and **HR**, reached **756.1**, **620.7** and **1018.0** **µM**, respectively. Additionally, 95% CI is shown in the Table 1 below. Expanded concentration range would allow more statistically significant IC50 values, but due to our study being only exploratory, such detailed analysis was off limits. Therefore, we’d like to encourage readers to treat those values more like a suggestion than a statement.

**Table 1:** Inhibitory concentration killing 50% of MCF7 breast cancer cells in the medium (both specific value and the range) observed for 3 most cytotoxic TFAx dipeptidomimetics in the dataset and statistical precision of the calculation (R squared), sorted by exact value of IC_50_, from most cytotoxic to the least.

Compound	IC_50_ (Best-fit values)	IC_50_ range (95% CI profile likelihood)	R squared (Goodness of Fit)
TFAx HV	620.7 µM	530.1 to 727.4 µM	0.9365
TFAx BAA	756.1 µM	652.1 to 881.3 µM	0.9317
TFAx HR	1018.0 µM	934.8 to 1152.0 µM	0.9394

**Fig. 5:**
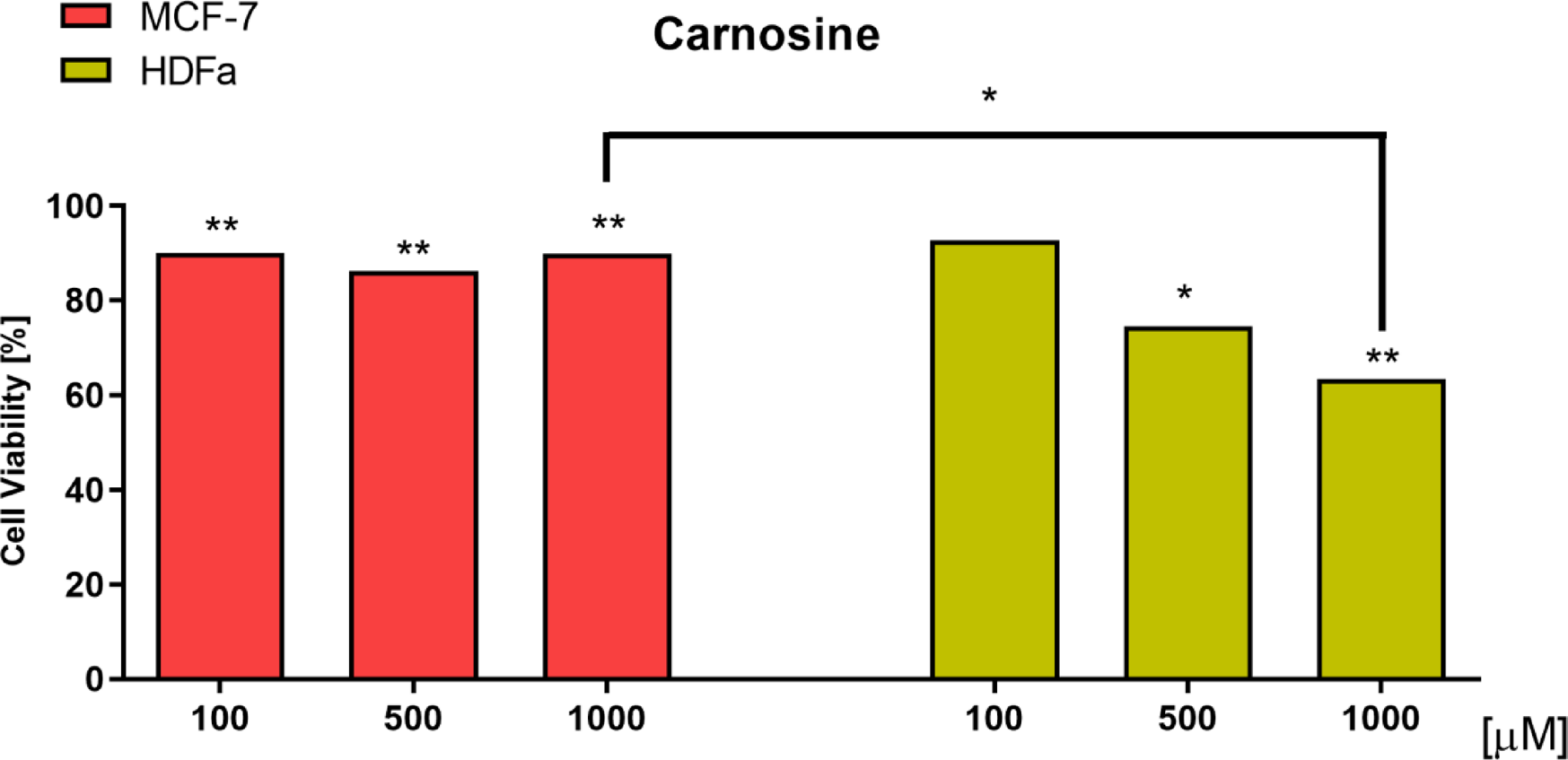
Cytotoxicity MTT test of carnosine on MCF7 (red bars) & HDFa (green bars) after 48 h of incubation. Mean ± SD was count based upon 3 independent experiments for each cell line; Tukey’s test: *p < 0.05; ***p < 0.001 Y axis represents percentage of viable cells, in comparison to untreated control (DMSO-treated cells, where DMSO did not exceed 0.5%).

Furthermore, Fig. 5 presents the results for carnosine’s cytotoxicity. MCF7 viability was impacted in a similar manner to the effect registered in the literature, even the slight increase of viability with increasing concentration—was also replicated^56^. However, a slightly more intense cytotoxic effect was observed for HDFa, rather than for MCF7. This might be related to the fact that carnosine has an anti-fibrotic effect, which can cause inhibition of collagen production, and decrease fibroblast activity within in vitro stressful setting^69–72^.

**Fig. 6:**
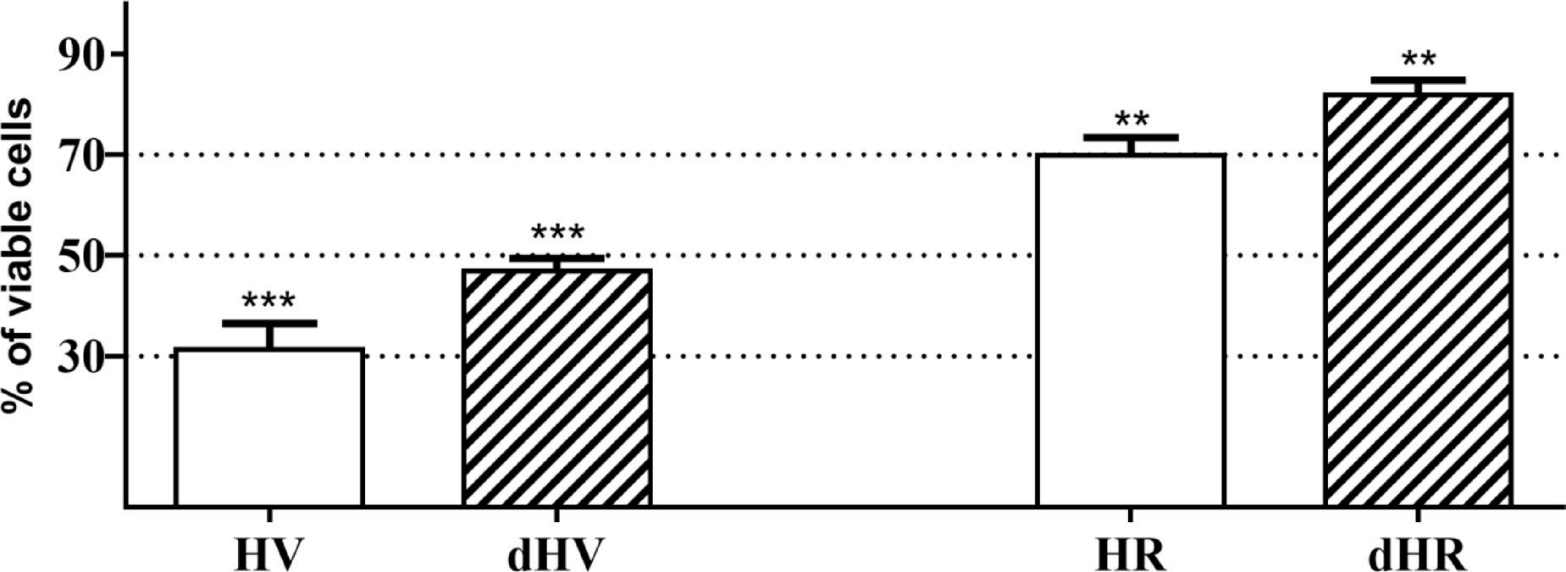
Cytotoxicity comparison between dipeptidomimetics with imidazole protected with benzyl group (TFAx **HV** and TFAx **HR**) and their unprotected derivatives **dHV**, **dHR** (stripped bars), both tested in 1 mM concentration in MCF-7 cells after 48 h of incubation, presented as mean ± SD from 3 independent experiments composed of 3 replicates. **p < 0.05, ***p < 0.001 vs control (untreated cells).

Next, two histidine dipeptidomimetics, **HV** and **HR**, had their imidazole residue deprotected to further analyze how it might impact their ability to decrease viability of MCF7 breast cancer cells. New derivatives, called **dHV** and **dHR**, respectively, were both found with about 25% less cytotoxicity compared to their protected equivalents (Fig. 6).

**Fig. 7:**
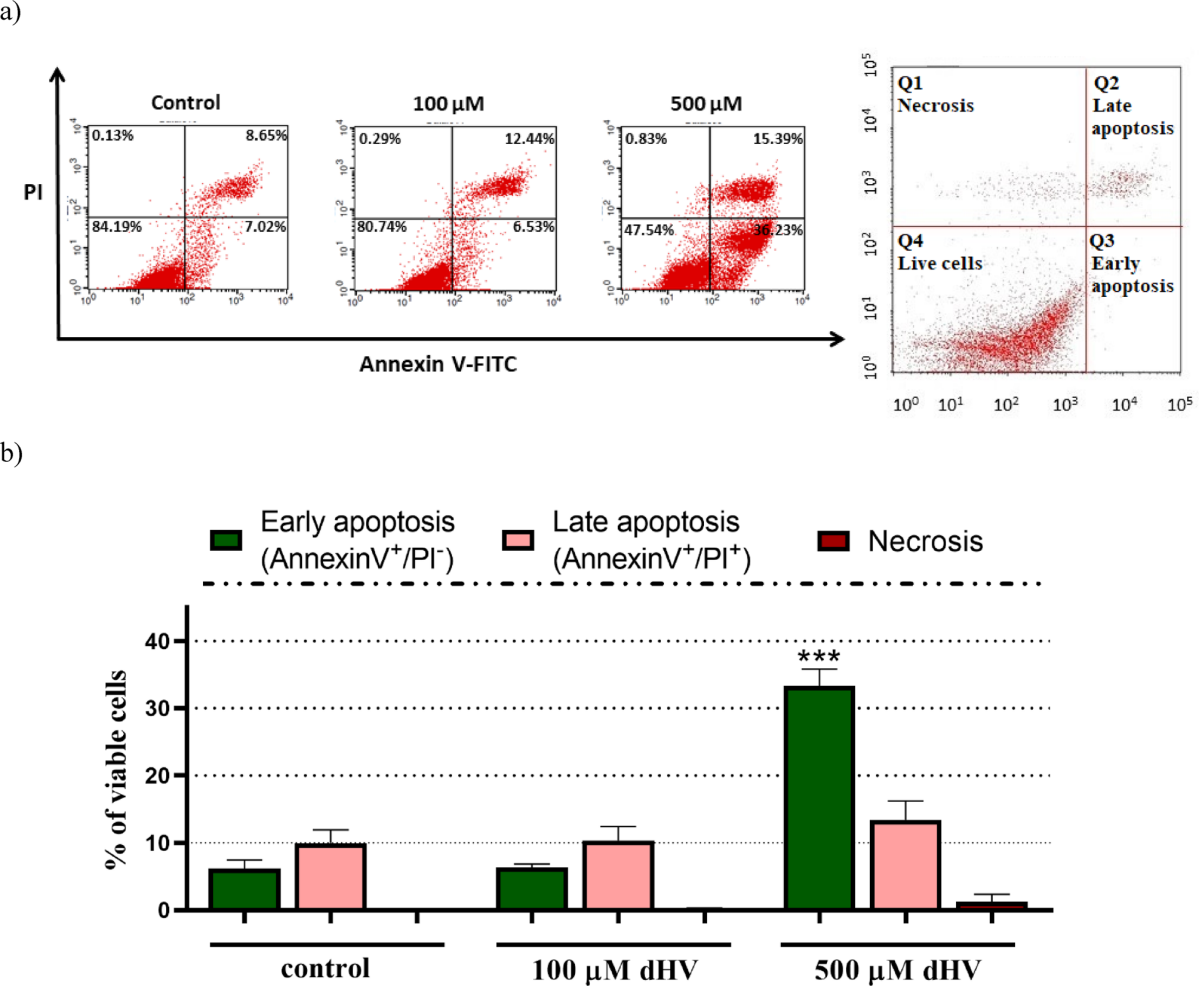
Detection of necrotic and apoptotic breast cancer MCF7 cells, after treatment with **dHR**. **a** 2D scatter plot representation of observed cells, with explanation of the graph components on the right; **b** bar graph with the percentage (%) of different observed cells (data presented as mean ± SD, from 3 separate experiments. **p < 0.01; ***p < 0.001 in relation to the control.

Due to the exploratory nature and reduced funds for this study, we were only able to test 1 compound for apoptosis. Flow cytometry was used to determine the percentage of cells in necrotic and apoptotic state after 48 h incubation of MCF7 breast cancer cells with **dHV** derivative, in 2 different concentrations: 100 and 500 μM. Results are presented in Fig. 7, through a bar graph and a scatterplot, including explanation of Q1-Q4 sections). Interestingly, in 500µM concentration **dHV** was found to put over 30% of MCF7 breast cancer cells into early apoptosis, without significant rise in necrotic cells.

### Computational studies

#### Target fishing

**Fig. 8:**
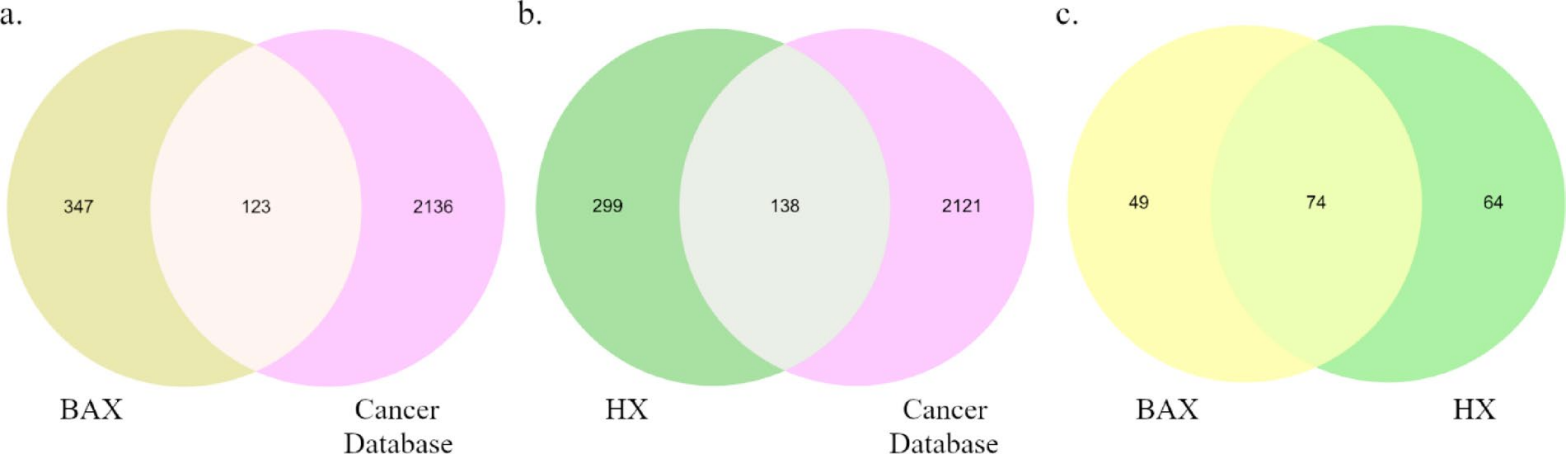
Venn diagrams presenting **BAX**/**HX** proportion of found cancer-related genes (**a**/**b** respectively) and a summarizing how many of those extracted genes overlap in both **BAX** and **HX** sets (**c**).

Two sets of dipeptidomimetics, TFAx **BAX** and TFAx **HX** were submitted to the PharmMapper inverse docking server. Results represented a list of proteins potentially impacted by input substance. This means that enzymes on the list might suffer a change in their metabolism upon treatment with our drug of choice. In total, 123/470 **BAX** & 138/437 **HX** impacted proteins were related to cancerogenesis, just like 138 out of 437 were matched for **HX** set. Sets **BAX** and **HX** were found to share more than 50% cancer-related attractive protein targets (Fig. 8).

UniProt IDs of found targets can be found in Table 1. in Supplementary Materials.

Lists of cancer enzymes acquired for **BAX** and **HX** were further uploaded to the STRING database to identify the functional partnerships and interactions between proteins, through PPI Network. This way we can explore what metabolic changes can our drug of interest relate to. Moreover, this method allows to define interesting targets for molecular docking. Table 2 in Supplementary Data summarizes number of nodes and edges of presented networks, with and without the “Degree > 9” cut-off. Even though the **HX** set comprises of a higher number of nodes in general, less of them survive the Degree cut-off than in **BAX** set. Figure 4 shows the network of most interesting proteins.

**Fig. 9:**
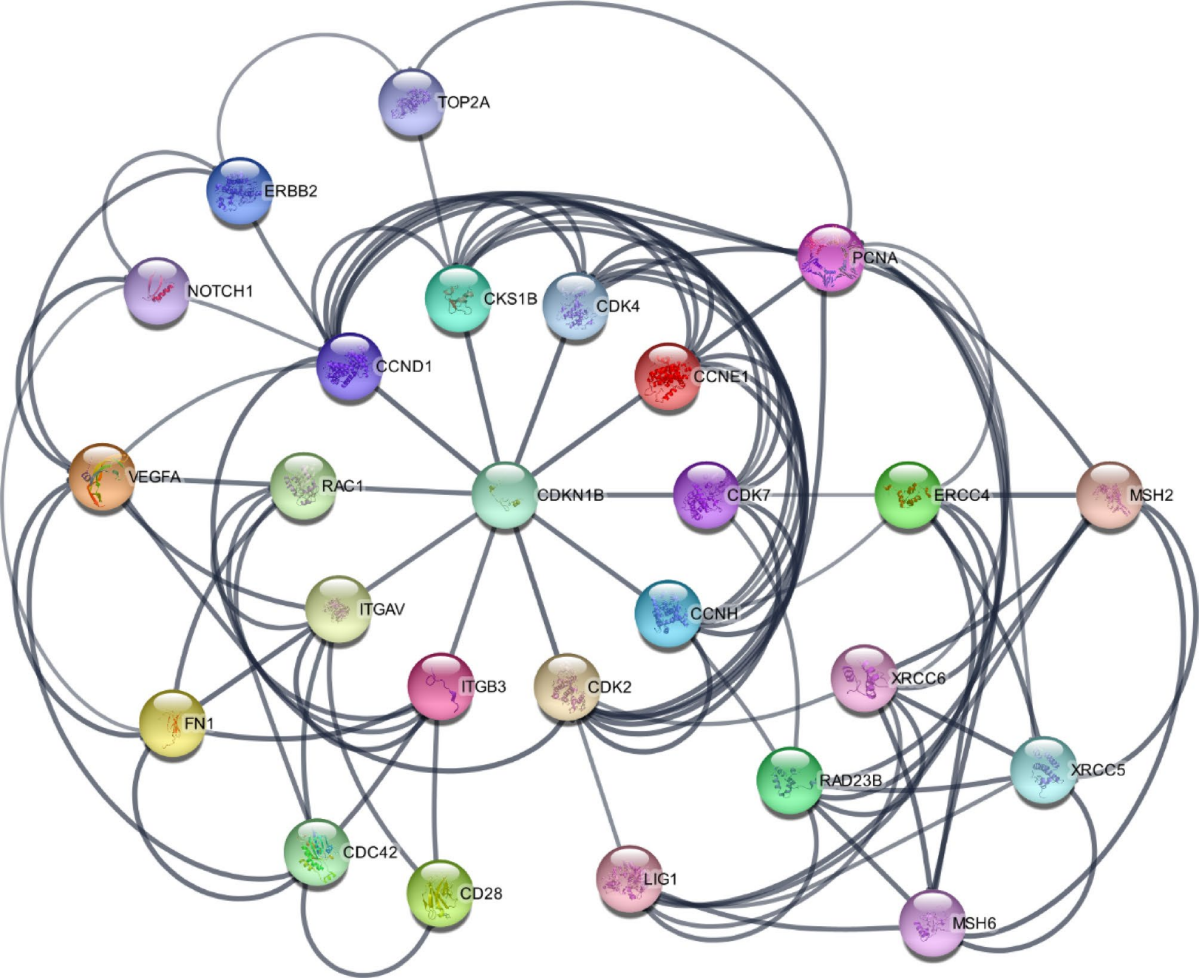
Protein–protein interaction network for the set **BAX**.

**Fig. 10:**
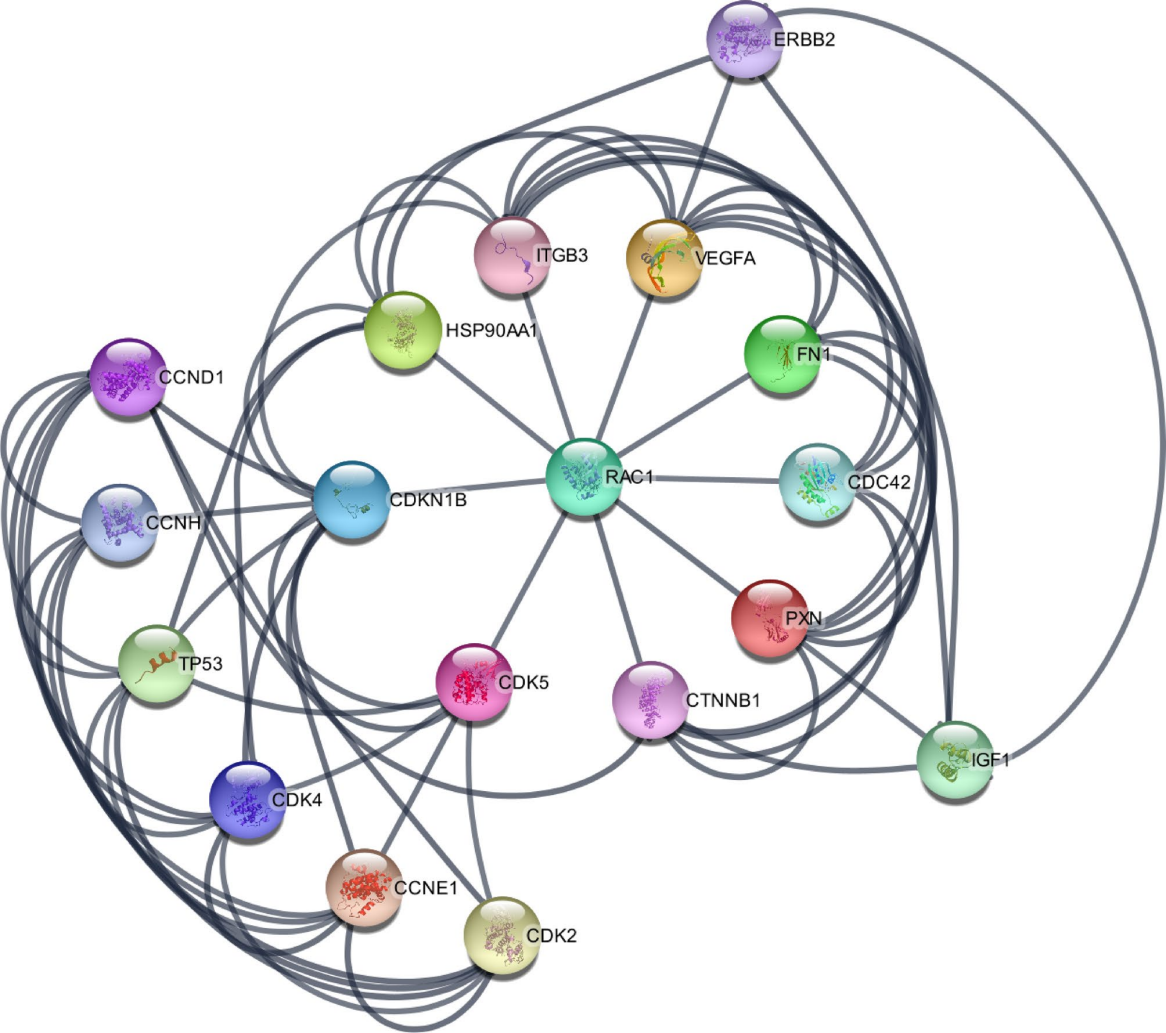
Protein–protein interaction network for the set **HAX.**

In order to put the importance of given nodes in a broader perspective, the networks are shown in Figs. 9 and 10, corresponding to the sets **BAX** and **HX**, respectively.

Both show Rac1 as one of the key targets, with the degree of 11 and 12, for **BAX** and **HX**, respectively. This enzyme, Ras-related C3 botulinum toxin substrate 1, represents one of the most studied members of Rho-GTPases family and is involved not only in cell cycle progression, but also with cytoskeleton reorganization, and its dysregulated expression had been found common in many types of malignancies, to mention breast or lung cancer, leading to tumor progression and metastasis^73–76^. Another common feature is VEGFA, referring to a key mediator of angiogenesis^77^, inhibited when Rac1 activity is hindered^78^, suggesting the ligands could impact cell growth through different routes. Similar tendency can be applied when investigating cyclin dependent kinases (CDKs), which are also affected by **BAX** and **HX** structures.

Common for both sets was also ERBB2, a growth factor found overexpressed in about 30% of diagnosed breast cancer patients, found disruptive and resulting in the rise of aggressive cancer cells, correlating to the severity of the disease^79^.

Herein from these examples it is visible that β-alanine and histidine dipeptides should affect cell cycle progression, specifically in G1 phase transitions. This consideration will be slightly discussed later in the results via biological experiments.

Investigating further, enzymes involved in the process of DNA binding and repair were also found present in the networks. Good example of such is topoisomerase IIα (TOP2A), essential for DNA repair system and already serving as a target for many anticancer drugs, like doxorubicin, amsacrine, etoposide and mitoxantrone. Given that peptides offer additional possibilities of interaction between the amino acids of a given enzyme and the ligand through hydrogen bonding, they might be found useful as complex partners for mentioned drugs, to secure the attachment—and maybe even define specificity, which in case of topoisomerase II would be very desirable.

Interestingly, PCNA, XRCC6, MSH2, XRCC5, ERCC4, MSH6 and LIG1 – also have the activity related to DNA, which might also stand as a clue that β-Alanyl dipeptides have the potential in interfering with DNA-related processes.

Through the analysis of **HX** PPI-Network, we can easily find further clues on the involvement of tested peptides in cell cycle progression, with targets like p53 or IGF1, involved in apoptotic pathways like PI3K/Akt, and Ras/MAPK. Building a PPI Network allowed us to explore possible interactions that **BAX** and **HX** dipeptidomimetics might acquire. Moreover, it helped define Rac1 as interesting and possibly important target for molecular docking experiments. Given that **dHV** was found to induce early apoptosis, we’ve decided to also dock our proposed dipeptidomimetics against a well-known mediator of apoptosis, caspase 3, which is also an established target for carnosine^80,81^. While all 20 proposed dipeptidomimetics show similar effect in molecular docking, it seems that those with deprotected imidazole, **dHV** and **dHR, s**eem to have slightly better energy of binding, although the difference is quite small. While most of the Cas3 scores for **BAX** and **HX** were of the same or better binding affinity than the one of carnosine, Rac1 binding was only found more efficient than carnosine only in the case of **BAV**, **BAF**, **HI**, **HF** and **HR** (Table 2).

**Table 2:** Free energies of binding [kcal · mol^-1^] obtained for the proteins Cas-3 and Rac1, for compounds **BAX**, **HX**,** HX2**, carnosine and MSI (isatin sulfonamide inhibitor cocrystalized with the X-ray structure of caspase-3, pdb id: 1GFW) and 1A-116 (taken from our validation for Rac1, pdb id: 1MH1).

Cas-3	BAX	HX	Rac1	BAX	HX
G	− 4.1	− 4.5	G	− 4.3	− 4.3
A	− 4.2	− 4.2	A	− 4.3	− 4.6
V	− 4.9	− 5.3	V	− 4.7	− 4.5
L	− 5.3	− 5.2	L	− 4.6	− 4.6
I	− 5.0	− 5.7	I	− 4.6	− 5.2
F	− 6.0	− 6.5	F	− 5.3	− 5.5
E	− 4.8	− 5.8	E	− 4.4	− 4.9
D	− 5.0	− 5.6	D	− 4.2	− 4.5
R	− 5.6	− 6.5	R	− 4.3	− 5.2
dHV	− 6.4		dHV		− 5.2
dHR	− 7.3		dHR		− 5.3
Carnosine	− 4.9		Carnosine		− 4.7
MSI	− 8.0		1A-116		− 6.6

#### Enrichment analysis

Targets from PPI Network with the Degree of at least 10 were retrieved as input (Table 3; Supplementary Data**)** for Enrichment Analysis, which means only proteins with at least 10 connections to other elements in the Network can be deemed important enough to be included in the further analysis. PPI Network can be difficult to read by someone who is not a biochemistry expert, and therefore labeling data based on the functionality of found enzymes can help clarify the medical potential, especially in the basic research state.

Enrichment is such a labeling method that helps us computationally classify the possible functionality and occurrence of potential drugs, just like a dictionary. Gene Ontology helped define possible biological processes (BP), cellular components (CC) and molecular functions (MF) that **BAX** and **HX** dipeptidomimetics could disrupt. Both sets seemingly interfere with G1/S transition of mitotic cell cycle, interrupt cell proliferation and affect protein maturation through binding phosphatases, kinases and Rho GDP. Those traits are also shared by both carnosine as well as paclitaxel, known anti-cancer drug; to initiate apoptosis^82^. All dipeptidomimetics seem to position themselves inside nucleoplasm.

KEGG Pathway Analysis classifies input enzymes to define possible pathway or metabolic route that might be disrupted. Most abundant results became „cell cycle”, „PI3K-Akt signaling pathway”, „pathways in cancer” and „proteoglycans in cancer” for both **BAX** and **HX**, which supports earlier hypothesis that those dipeptidomimetics could potentially be useful against cancer.

**Fig. 11:**
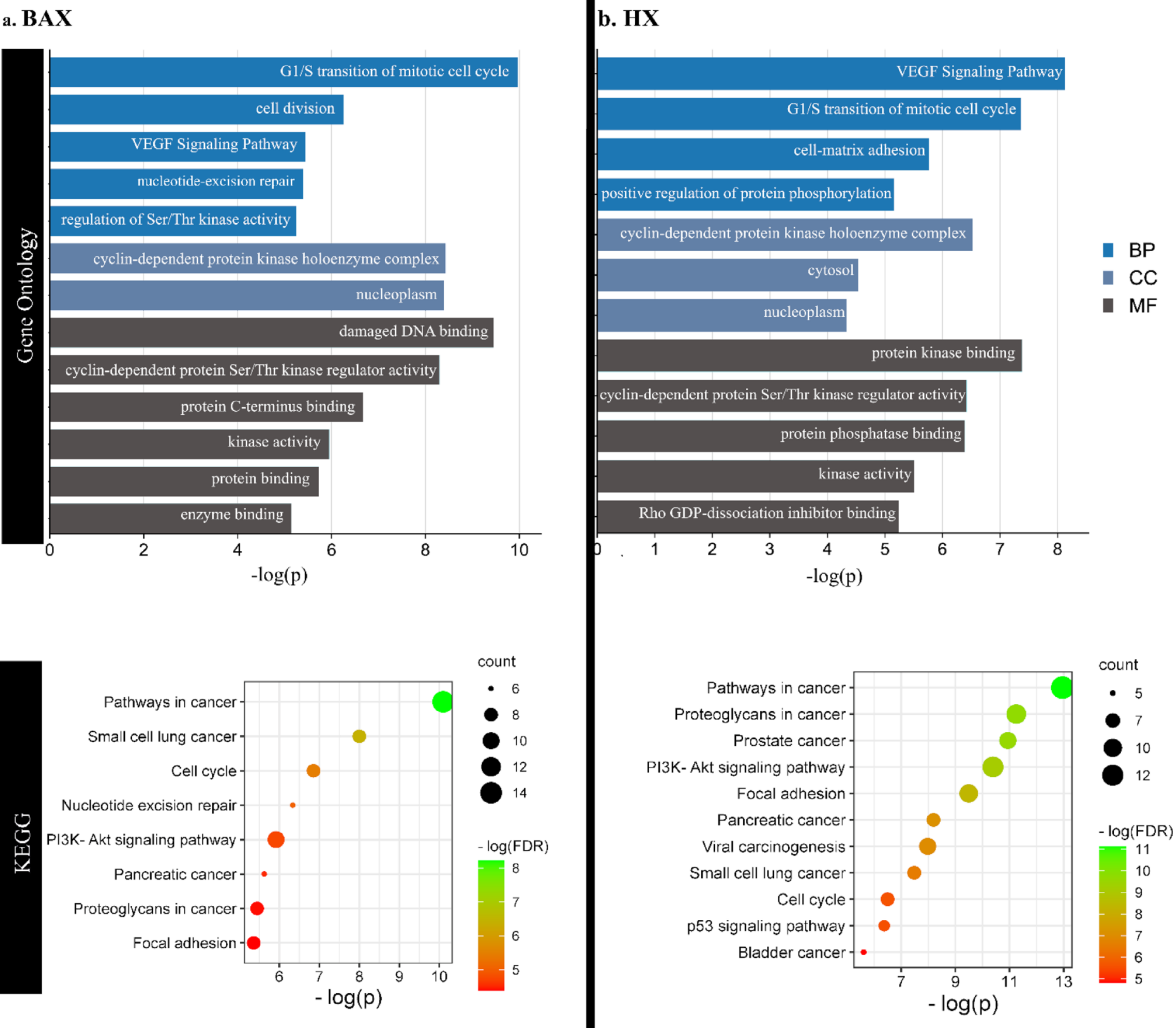
Graphical representation of the most statistically significant and important variables obtained for gene ontology enrichment (BP: biological process, CC: cellular component and MF: molecular function) and KEGG for sets **BAX** and **HX** (**a** and **b** respectively). KEGG reference dataset was prepared by Minoru Kanehisa^83^.

Raw data can be found in Tables 4–7 in Supplementary Data, in case other researchers would like to further inspect those results, although their most important outcomes are visualized in Fig. 11 graphs.

#### ADME

For the sake of ADME analysis, 20 dipeptidomimetics, earlier discussed in biological studies, with free *N*-terminal, without consideration of TFA ion, were prepared in the .smi and.mol2 format.

Theoretical assessment of metabolism (ADME) helps trace metabolism of proposed drugs before their our compounds of interest and assess potential drug-likeness. Although peptides have their own transporters and dedicated hydrolyses – ADME methodology is efficiently incorporated for small molecules and peptides, as they bring useful perspective to the overall potential for oral bioavailability^84–88^. While this aim is not perfect and might suffer differences to the in vitro found catabolism—it still offers a general view which can support and expend the view on the presented cytotoxicity results.

We found all dipeptidomimetics to pass the Lipinski’s Drug-likeness rule for oral drug distribution, although arginine-related derivatives (TFAx BAR/HR/dHR) were characterized with a low probability of absorption through gastrointestinal tract, due to TPSA (Topological Polar Surface Area) value too high to facilitate successful oral drug administration^89^. Further exploring solubility patterns, histidine-based compounds were found proportionally more attractive to lipids, compared to beta-alanine derivatives (therefore bearing higher logP values and lower solubility in water).

**Fig. 12:**
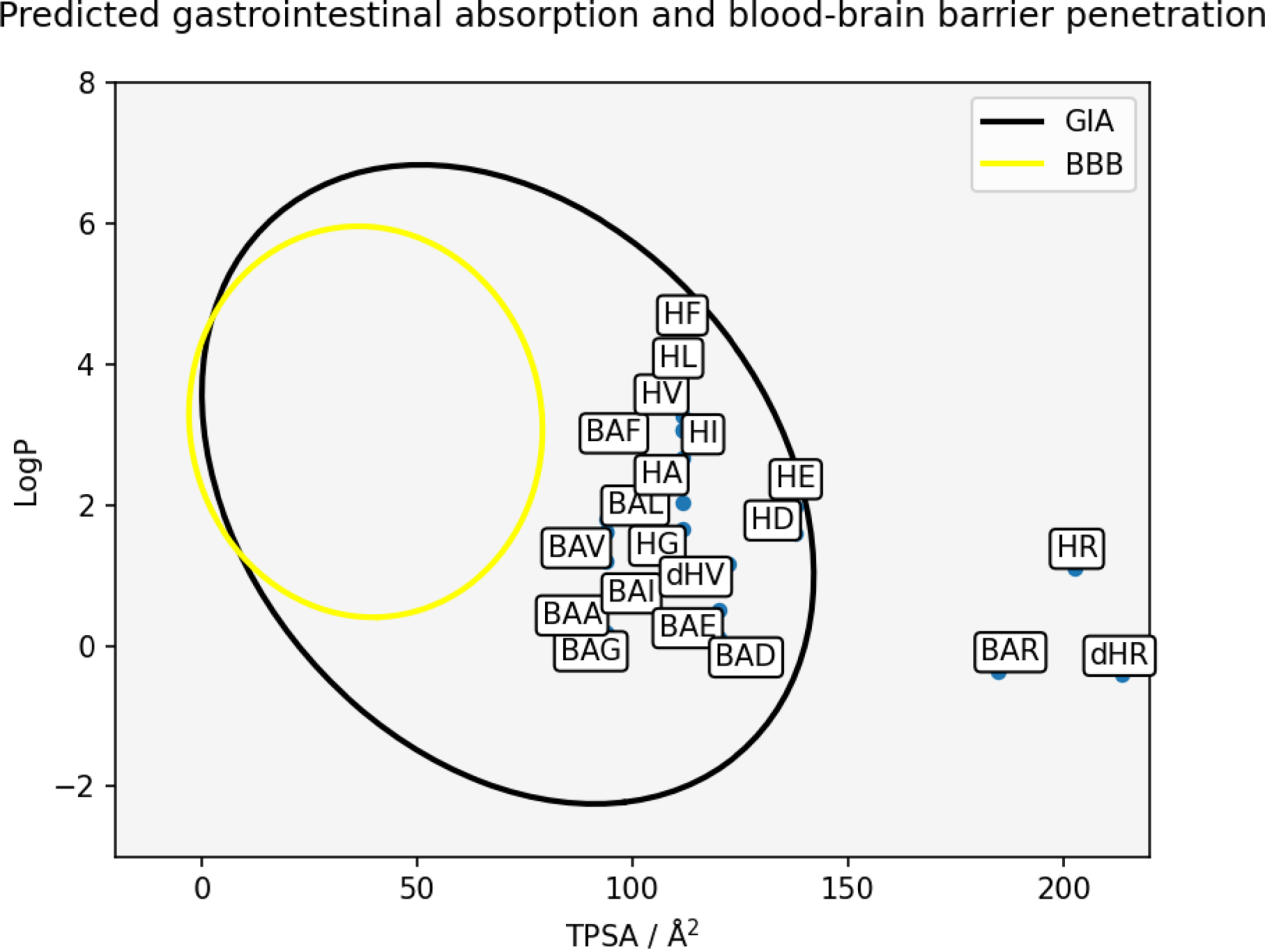
BOILED-Egg graph visualizing theoretical estimation of oral consumption efficacy 20 considered dipeptidomimetics (unlabeled points are arginine derivatives TFAx BAR, HR and dHR). Circles represent possible gastrointestinal absorption (GIA) and fitness to pass the blood–brain barrier penetration cut-off enabeling the drug to reach the brain (BBB).

**BAA** was found 5 times more soluble in water than **HV**, translating to potentially higher efficacy via oral administration . Given that solubility is very important for the therapeutic index of a supposed drug—a hypothesis might be drawn that short oligopeptides with β-alanine constituents might pose to be more attractive for medical purposes, than those of histidine. Moreover, β-alanine derivatives had higher QED (structural similarity to drugs already on the market), despite substantially lower molecular weight (MW), as well as number of rotatable bonds and heavy atoms, in comparison to histidine. Interestingly, imidazole deprotection reduced the LogP value of **HV** almost to zero, which suggests higher solubility in water. The same change was observed between **HR** and **dHR** but to a smaller extent. Generally most of the proposed dipeptidomimetics were predicted to be able to pass the gastrointestinal tract, and therefore potentially could be administered by oral route. However, caution must be taken, because enzymatic hydrolysis can potentially play a role in changing those predispositions in vivo. Interestingly, ADME positions **HR** as less relevant than **HV** or **BAA**, due to possible solubility problems. Moreover, this additional method has shed some new light on our data, allowing to see that both protection as well as deprotection can be a useful tool to steer the physicochemical properties of our drug of interest. Last but not least, while **dHV** was found with less cytotoxicity than **HV** over MCF7 breast cancer cell line, it seems that this increase in polarity can actually be a successful tactic to design new cancer-targeting conjugation partners from dipeptidomimetics. However, this might also suggest that instead of deprotecting imidazole, conjugating the chain with a toxic, but well known cancer drug, could benefit from free imidazole, as well as additional positive charge. Nevertheless, on the basis of presented exploratory data, this hypothesis is yet to be determined (Fig. 12).

Full ADME data is shown in Supplementary Data, Table 8, while its visualization is presented in the main manuscript in a form of a BOILED-Egg graph, in Fig. 8.

## Discussion

Cancer malignancies need a plethora of resources to grow and reach metastasis. Leucine withdrawal has been found to help silence the immune response during cancerogenesis^90^, and can disrupt glutamine, asparagine, aspartate, histidine, arginine and serine metabolism^91–93^. In fact, arginine is so extensively recruited by cancer cells that some researchers suggest arginine deprivation therapy could be attractive as a part of medical treatment^94^.

Breast cancer is one of the most abundant malignancies worldwide, and recently peptide-based therapeutics had been tested against it to broader extent^95–97^. Although such experiments usually take a lot of time and finances, computational methods offer additional insight into the screening phase, with the ability to cross validate results between in silico and in vitro.

Metabolomics of breast cancer had recently revealed leucine, isoleucine, glutamine, methionine, valine, serine and histidine are up taken by tumor cells (HCC 1806) whereas human breast epithelial cell line MCF10A did not. Moreover, alanine, cysteine, aspartic and glutamic acids were observed to be excreted outside the cell^91^. While glutamic acid plays an equally important role in amino acid metabolism as leucine, the general hypothesis to such export is mentioned compounds are intermediate metabolites excessively overproduced through proliferation-related enzymatic cascades, however having a major impact on the neighboring healthy cells^98^. Moreover, high concentration of both glutamic and aspartic acid were found to inhibit tumor proliferation rate, which might serve as another clue on why the cancerous cell excretes them^99,100^. Additionally, a broader computational analysis had shown the highest difference in metabolite concentration between healthy and cancerous breast cells to be represented by glutamic acid, followed by β-alanine and serine. This might suggest that those amino acids are more prevalently attracted towards cancer cells and therefore might serve as transporters for toxic drugs, like cisplatin. Contrary to glutamic acid—β-alanine is accumulated in tumor tissues, rather than excreted^3^, hence suggesting its higher importance in cancer expansion. Indeed, our cytotoxicity results show that dipeptidomimetics with beta-alanine were generally more successful in reaching cytotoxicity towards MCF7 cells. Such observation was especially interesting, considering that most of the proposed dipeptidomimetics were found more effective than carnosine itself. Carnosine is generally thought to be 50% less stable than β-alanine, but 10-times higher than L-histidine^101^. Moreover, long β-alanine supplementation is suggested to reduce the blood concentration of histidine, and such deficiency can lead to silencing the immune response to cancer and faster tumor growth^102,103^. This could be an additional limitation for beta-alanine derivatives in cancer therapy, although it didn’t seem to occur in our in vitro studies.

Higher beta-alanine concentration also makes the pH in the cell more alkaline, which correlates with further inhibition of glycolysis– in both cancerous and normal breast cells (MCF7 and MCF10A, respectively). Additionally, ATP concentration was declined in MCF7, whereas in MCF10A—it became elevated, suggesting different energy metabolism patterns^57,104^. Given the impact of **BAX** and **HAX** on kinase binding, cell division and nucleic acid binding, those derivatives could potentially also disrupt mitochondrial energy production. This is also suggested due to an abundant amount of attractive enzymatic targets to be related to mitosis and PI3K pathway.

Moreover, anti-angiogenic potential of His-Gly and His-Phe dipeptide-zinc complexes has already been established, with proposed ability to intercalate and block enzymes related to nucleic acids, one of which is telomerase, responsible for cancer immortality^8,105^. Although neither **HG** nor **HF** presented themselves with substantial cytotoxicity over MCF7, they did show a good docking score to caspase 3 and Rac1, which also partially depend on nucleic acids synthesis.

Generally, any peptide with a modification on its constituent amino acids backbone, with the pursuit of enhanced medical functionality or extended stability—can be called a peptidomimetic^106–110^. Most important outcome of such derivation is to make the compound unrecognizable to exopeptidases, which would normally target either *N*- or *C*-terminal amino acid in the peptide chain, which can even be obtained through simple esterification. Moreover, such pursuit can deeply improve kinetic properties of the compound, especially when the amino acid in question constitutes of charged polar group, as protection of C-terminal results in enhanced hydrophobicity and overall improved metabolic stability, of what L-NAME, a methyl esther nitroarginine derivative, is a very good example^93,111^.

For example, while studying the cytotoxicity of a tetrapeptide MMAF (methionine-methionine-alanine-phenylalanine), it was found that if *C*-terminal phenylalanine residue was protected with a methoxy group, resulting in an ester—the charge reduced to 0, whereas IC_50_ substantially dropped and overall enzymatic activity was more than 2 thousand times more effective^112^. Furthermore, carboxylic acid derivative CA-074 (similar to peptides in shape and size) is unable to pass the cell membrane barrier, while its methyl ester analog sufficiently gets inside the cancer cell and inhibits cathepsin B, protein deeply overexpressed in many tumor types, leading to autophagy of neighboring healthy cells^111^. Such logic made us consider exploring the difference with **HV** and **HR**, compared to **dHV** and **dHR**, respectively. Free imidazole might result in higher polarity, which could potentially make cell entry more difficult, though more studies would need to be performed to prove it. Moreover, positive charge changes physicochemical properties of the drug. Smaller prevalence of histidine-related derivatives (rather than beta-alanine ones) to cause cytotoxicity in MCF7 breast cancer cells might therefore come out of worse structural properties, rather than functionality. However, more studies should follow to either support or discredit this suggestion.

Our study sheds more light onto the applicability of *C*-terminal methyl ester peptidomimetics, and protected peptide derivatives in general. We’ve also proposed inclusion of computational methods in exploratory search for medical substances, as a cost-saving, insightful way to suggest further explanation of acquired biological results. TFAx **HV** (rather than TFAx **BAA)**, was found with higher cytotoxicity over MCF7 breast cancer cells from the proposed substances, although beta-alanine was reportedly a good conjugate linker (aliphatic carbon chain), which might suggest that dipeptidomimetic TFAx **BAA** could be most attractive based on the abilities it acquires upon conjugation, rather than on its own.

For an extensive understanding of this topic, research with a wider variety of in vitro cell lines and expanded concentration range would be necessary to further ensure true functionality of proposed dipeptidomimetics. This study serves as an exploratory screening with the aim to interest other scientists in further investigation, although it could itself be developed into extensive and detailed analysis. However, considering the abundance of peptide combinations, simple exploration of potential mimetics can be a useful starting point in the drug design process. Furthermore, more cell lines could be used to ensure observed cytotoxicity was replicable and consistent. Although MCF-7 line is one of the most abundant in breast cancer research, it cannot serve as the sole reasoning for the anti-cancer effect. Breast cell lines like MDA-MB-23 and BT-474 (cancerous), or MCF10A (healthy), as well as broader drug concentration range, would be advised to further expand presented data. Our preliminary study only serves as guidance, showing potential of small peptide derivatives in cancer treatment. Nevertheless, it’s important to consider that the properties found for dipeptidomimetics might change upon conjugation to anti-cancer drug of choice.

Last but not least, we’d like to emphasize how many more hypotheses and ideas were drawn thanks to the involvement of computational studies. Although prone to errors, they can be a compass, steering our focus and attention towards most attractive derivatives. Such theoretical experiments are of even higher importance when we consider that physicochemical, just like functional activities can change upon conjugating peptidomimetics with other medical substances—and in such situations computational data becomes even more important. We hope that our modest experiments present a decent example of how computational power can improve our knowledge during research. We deeply believe that theoretical chemistry in drug design research can offer much more data validation before reaching clinical trials stage, a step which more than 90% candidates later fail^113^.

## Conclusions

Although dipeptides are generally not very toxic compounds, their mimetics can help with cancer drug transport directly to the healthy cells. They seem to be an attractive starting point for conjugates or complexes, especially due to their low stability. Two TFAx dipeptidomimetics were found with IC_50_ lower than 1 mM: **HV** and **BAA**, with 756.1 and 620.7 µM, respectively. Although high, these values do not come far from their natural peptide derivatives, and could still prove themselves useful upon conjugation with a toxic cancer drug, like cisplatin or fluorouracil. Computational data suggests both beta-alanine and histidine related peptidomimetics can impact cancer metabolism and disrupt clinically relevant pathways. Moreover, cell division seems to be key process affected by the compounds, which increases their medical potential. Although selectivity observed in biological studies was substantial, it still deserves recognition, as it could suggest the ability to increase selectivity of conjugation partners.

It is very important to note, that carnosine (a dipeptide composed of β-Ala-His) didn’t disrupt MCF7 proliferation even in 1000 µM concentration (1mM), while our proposed dipeptidomimetics did^56^. What is more, TFAx **BAA** (**β-Ala-Ala**) seems to possess much broader impact on MCF7 than carnosine. This observation challenges the long accepted paradigm of L-histidine being the main reason for carnosine effectivity, although we cordially invite all experts from the field to elaborate. Moreover, we’d like to emphasize on the potential of beta-amino acid peptidomimetics their enhanced stability, attractiveness as conjugation partners and possible impact on the metabolism of already existing drug^37^. Peptidomimetics represent a huge amount of possible compounds, and using interdisciplinary methods to understand their potential before proper synthesis—is advised, as a measure to increase research effectivity, without the need for additional funding. Our study represents an example, which we hope to be inspiring for experts.

## Materials and methods

### Synthesis

Thin-layer chromatography (TLC) and preparative thin-layer chromatography (PTLC) were performed on silica gel 60 F254 plates purchased from Sigma Aldrich. Column chromatography was conducted using silica gel 60 (70–230 mesh), Sigma Aldrich. ^1^H NMR, ^13^C NMR data were obtained on a Varian Unity 500 Plus spectrometer (500 MHz for ^1^H, 101 MHz for ^13^C). Chemical shifts (d) are reported in ppm relative to the residual peak of chloroform as a solvent (^1^H 7.26 ppm and ^13^C 77.16 ppm). A total proton decoupling was applied for ^13^C NMR spectra. For ^1^H NMR description, the following symbols are used: s (singlet), bs (broad singlet), d (doublet), t (triplet), q (quartet), quint (quintet), sext (sextet), sept (septet), m (multiplet); and the coupling constant values (*J*) are determined in Hertz. Mass spectra were measured using Agilent 6470 Triple Quadrupole Analyzer, a LC/MS system with ESI (Electrospray Ionization Source). This equipment was funded by the Foster Foundation (USA) and we are deeply grateful for the opportunity and scientific help that they provided us with.

### Protected dipeptidomimetics

*General procedure*. Boc-β-Ala-OH or Boc-His(Bn)-OH (0,65 mmol) was dissolved CDI (0.793 mmol) in anhydrous DCM (30 ml). After 45 min, a solution of a chosen amino acid methyl ester hydrochloride (0.793 mmol) solution with DCM (30 ml) and DIPEA (0.793 mmol) was added to the mix and stirred at room temperature on a magnetic centrifuge for 24 h. The progress of the reaction was monitored by TLC in a CHCl_3_:MeOH (9:1) system. After distilling off the solvent in vacuum, the crude products were purified by liquid chromatography (SiO_2_) and on preparative TLC plates in a system CHCl_3_:MeOH (9:1, *v/v*).

#### Boc-β-Ala-Ala-OMe

Yield: 48.51%.

^1^H NMR (500 MHz, CDCl_3_) δ ppm: 6.20 (s, 1H, NH), 5.19 (s, 1H, NH), 4.60 (m, 1H, CH), 3.78 (s, 3H, COOCH_3_), 3.43 (m, 2H, CH_2_), 2.46 (t,* J* = 5.9 Hz, CH_2_), 1.45 (s, 9H, CH_3_-Boc), 1.43 (d, *J* = 7.2 Hz, 3H, CH_3_).

^13^C NMR (101 MHz, DMSO-*d*_*6*_) δ_C_: 173.41 (C15), 171.08 (C3), 156.07 (C12), 79.33 (C7), 52.50 (C19), 48.02 (C16), 36.56 (C2), 36.13 (C1), 28.39 (C8, C9, C10), 18.28 (C14).

MS: *m/z* calculated for C_12_H_22_N_2_O_5_ 297.31, found 297.1 [M+Na]^+^.

#### Boc-His(Bn)-Val-OMe

Yield: 73.47%.

^1^H NMR (500 MHz, CDCl_3_) δ ppm: 7.60 (s, 1H, CH), 7.42–7.15 (m, 5H, Ph), 6.77 (s, 1H, CH), 6.39 (s, 1H, NH), 5.06 (m, 2H, CH_2_), 4.44 (m, 2H, CH), 3.70 (s, 3H, COOCH_3_), 3.04 (m, 2H, CH_2_), 2.10 (m, 1H, CH), 1.47 (s, 9H, CH_3_-Boc), 0.93 (d, *J* = 6.4 Hz, 6H, CH_3_).

^13^C NMR (101 MHz, DMSO-*d*_*6*_) δ_C_: 172.02 (C27), 171.56 (C16), 155.71 (C18), 137.88 (C4), 135.18 (C12), 129.12 (C2, C6), 128.60 (C3, C5), 127.68 (C1), 117.47 (C8), 77.34 (C21), 57.39 (C26), 51.97 (C14), 51.26 (C33), 50.83 (C7), 30.99 (C30), 30.15 (C13), 28.32 (C22, C23, C24), 18.88 (C31), 17.69 (C32).

MS: *m/z* calculated for C_24_H_34_N_4_O_5_ 459.55, found 459.3 [M+H]^+^.

#### Boc-β-Ala-Gly-OMe

Yield: 40.31%.

^1^H NMR (500 MHz, CDCl_3_) δ ppm: 6.20 (s, 1H, NH), 5.20 (s, 1H, NH), 4.07 (d,* J* = 5.3 Hz, 2H, CH_2_), 3.79 (s, 3H, COOCH_3_), 3.45 (m, 2H, CH_2_), 2.49 (t, *J* = 5.9 Hz, 2H, CH_2_), 1.45 (s, 9H, CH_3_-Boc).

^13^C NMR (101 MHz, DMSO-*d*_*6*_) δ_C_: 171.69 (C8), 170.29 (C3), 156.04 (C17), 79.42 (C13), 52.44 (C11), 41.20 (C7), 36.64 (C2), 36.11 (C1), 28.39 (C14, C15, C16).

MS: *m/z* calculated for C_11_H_20_N_2_O_5_ 283.29, found 283.1 [M + Na]^+^.

#### Boc-β-Ala-Val-OMe

Yield: 46.48%.

^1^H NMR (500 MHz, CDCl_3_) δ ppm: 6.08 (s, 1H, NH), 5.18 (s, 1H, NH), 4.57 (dd, *J* = 8.7, 5.0 Hz, 1H, CH), 3.77 (s, 3H, COOCH_3_), 3.43 (m, 2H, CH_2_), 2.49 (m, 2H, CH_2_), 2.17 (m, 1H, CH), 1.45 (s, 9H, CH_3_-Boc), 0.95 (m, 6H, CH_3_).

^13^C NMR (101 MHz, DMSO-*d*_*6*_) δ_C_: 172.42 (C15), 171.46 (C11), 156.02 (C8), 79.40 (C3), 57.10 (C14), 53.42 (C21), 36.79 (C10), 36.27 (C9), 31.15 (C18), 28.39 (C4, C5, C6), 18.94 (C19), 17.82 (C20).

MS: *m/z* calculated for C_14_H_26_N_2_O_5_ 325.37, found 325.2 [M + Na]^+^.

#### Boc-β-Ala-Leu-OMe

Yield: 47.50%.

^1^H NMR (500 MHz, CDCl_3_) δ ppm: 8.23 (d, *J* = 7.6 Hz, 1H, NH), 6.68 (t, *J* = 5.4 Hz, 1H, NH), 4.24 (m, 1H, CH-L2), 3.59 (s, 3H, COOCH_3_), 3.08 (m, 2H, CH_2_-A3), 2.25 (m, 2H, CH_2_-A2), 1.59 (m, 1H, CH-L4), 1.50 (m, 1H, CH-L3A), 1.43 (m, 1H, CH-L3B), 1.35 (s, 9H, C(CH_3_)_3_), 0.86 (d, *J* = 6.6 Hz, 3H, CH_3_-L5), 0.81 (d, *J* = 6.5 Hz, 3H, CH_3_-L6).

^13^C NMR (101 MHz, DMSO-*d*_*6*_) δ_C_: 173.54 (C–CO), 171.01 (C–CO), 155.86 (C-CO_Boc_), 78.02 (C–C _Boc_), 52.22 (C-OCH_3_), 50.56 (C-L2), 40.12 (C-L3), 37.04 (C-A2), 35.76 (C-A3), 28.65 (C-CH_3Boc_), 24.65 (C-L4), 23.18 (C-L5), 21.68 (C-L6).

MS: *m/z* calculated for C_15_H_28_N_2_O_5_ 339.39, found 339.2 [M+Na]^+^.

#### Boc-β-Ala-Ile-OMe

Yield: 47.84%.

^1^H NMR (500 MHz, CDCl_3_) δ ppm: 6.12 (s, 1H, NH), 5.19 (s, 1H, NH), 4.53 (m, 1H, CH), 3.76 (s, 3H, COOCH_3_), 3.43 (m, 2H, CH_2_), 2.47 (m, 2H, CH_2_), 1.89 (m, 1H,CH), 1.45 (s, 9H, CH_3_-Boc), 1.18 (m, 2H, CH_2_), 0.93 (m, 6H, CH_3_).

^13^C NMR (101 MHz, DMSO-*d*_*6*_) δ_C_: 172.44 (C15), 171.10 (C11), 156.01 (C8), 79.40 (C3), 56.40 (C18), 52.04 (C14), 37.70 (C14, C10), 36.08 (C9), 28.33 (C4, C5, C6), 23.06 (C21), 14.77 (C20), 11.47 (C22).

MS: *m/z* calculated for C_15_H_28_N_2_O_5_ 339.39, found 339.2 [M+Na]^+^.

#### Boc-β-Ala-Phe-OMe

Yield: 37.12%.

^1^H NMR (500 MHz, CDCl_3_) δ ppm: 7.30 (m, 2H, Ph), 7.12 (m, 3H, Ph), 6.08 (s, 1H, NH), 4.89 (m, 1H, CH), 3.75 (s, 3H, COOCH_3_), 3.38 (m, 2H, CH_2_), 3.13 (m, 2H, CH_2_), 2.39 (m, 2H, CH_2_), 1.46 (s, 9H, CH_3_-Boc).

^13^C NMR (101 MHz, DMSO-*d*_*6*_) δ_C_: 171.95 (C15), 171.11 (C11), 155.98 (C8), 135.77 (C18), 129.19 (C19, C23), 128.65 (C20, C22), 127.21 (C21), 79.34 (C3), 53.12 (C14), 52.41 (C25), 37.90 (C16), 36.59 (C10), 36.16 (C9), 28.33 (C4, C5, C6).

MS: *m/z* calculated for C_18_H_26_N_2_O_5_ 373.18, found 373.2 [M+Na]^+^.

#### Boc-β-Ala-Glu-(OMe)_2_

Yield: 50.25%.

^1^H NMR (500 MHz, CDCl_3_) δ ppm: 6.40 (s, 1H, NH), 5.26 (s, 1H, NH), 4.56 (m, 1H, CH), 3.77 (s, 3H, COOCH_3_), 3.70 (s, 3H, COOCH_3_), 3.43 (m, 2H, CH_2_), 2.44 (m, 4H, CH_2_), 2.23 (m, 1H, CH), 2.02 (m, 1H, CH), 1.45 (s, 9H, CH_3_-Boc).

^13^C NMR (101 MHz, DMSO-*d*_*6*_) δ_C_: 173.11 (C20), 172.20 (C15), 171.07 (C11), 156.02 (C8), 79.47 (C3), 60.16 (C4), 52.37 (C24), 51.53 (C23), 36.58 (C10), 35.92 (C9), 29.98 (C19), 28.26 (C4, C5, C6), 26.93 (C16).

MS: *m/z* calculated for C_15_H_26_N_2_O_7_ 369.38, found 369.2 [M+Na]^+^.

#### Boc-β-Ala-Asp-(OMe)_2_

Yield: 42.80%.

^1^H NMR (500 MHz, CDCl_3_) δ ppm: 6.62 (s, 1H, NH), 4.89 (m, 1H, CH), 3.79 (s, 3H, COOCH_3_), 3.72 (s, 3H, COOCH_3_), 3.48 (m, 2H, CH_2_), 3.05 (m, 1H, CH), 2.86 (m, 1H, CH), 2.47 (m, 2H, CH_2_), 1.43 (s, 9H, CH_3_-Boc).

^13^C NMR: 171.50 (C11), 171.30 (C20), 171.08 (C15), 155.94 (C8), 79.48 (C3), 52.69 (C18), 51.95 (C23), 48.39 (C14), 36.92 (C19), 36.59 (C10), 35.97 (C9), 28.28 (C4, C5, C6).

MS: m/z calculated for C_14_H_24_N_2_O_7_ 355.35, found 355.2 [M+Na]^+^.

#### Boc-β-Ala-Arg(NO_2_)-OMe

Yield: 45.64%.

^1^H NMR (500 MHz, CDCl_3_) δ ppm: 7.04 (s, 1H, NH), 5.30 (s, 1H, NH), 4.62 (m, 1H, CH), 3.77 (s, 3H, COOCH_3_), 3.41 (m, 4H, CH_2_), 2.53 (m, 2H, CH_2_), 1.91 (m, 1H, CH), 1.67 (m, 3H, CH, CH_2_), 1.43 (s, 9H, CH_3_-Boc).

^13^C NMR (101 MHz, DMSO-*d*_*6*_) δ_C_: 172.52 (C11, C15), 159.39 (C21), 156.34 (C7), 79.35 (C2), 52.74 (C14), 51.28 (C28), 40.59 (C19), 36.89 (C10), 36.35 (C9), 29.80 (C17), 28.39 (C3, C4, C5), 24.60 (C18).

MS: *m/z* calculated for C_15_H_28_N_6_O_7_ 427.42, found 427.1 [M+Na]^+^.

#### Boc-His(Bn)-Gly-OMe

Yield: 45.19%.

^1^H NMR (400 MHz, CDCl_3_) *δ 8.04 (s, 1H, C-(N)*_*2*_*-imi), 7.49–7.17 (m, 6H, Ph), 6.87 (s, 1H, NH), 6.10 (s, 1H, NH), 5.17 (q, J* = *34.3 Hz, 1H, CH), 4.89 (s, 1H, CH*_*2*_*), 4.64 (d, J* = *7.4 Hz, 2H, CH), 4.33 (s, 1H, 2xCH*_*2*_*), 4.00 (ddd, J* = *42.3, 18.0, 5.6 Hz, 2H, CH), 3.73 (s, 3H, CH*_*2*_* 1.41 (s, 9H, CH*_*3*_*-Boc).*

^13^C NMR (101 MHz, CDCl_3_) *δ 171.15 (s, CO-BA, CO-G, CO-Boc), 131.19 – 124.87 (m, C-Bn, 1xC-imi), 77.82–74.66 (m, C(CH*_*3*_*)-Boc), 60.40 (s, OCH*_*3*_*-G), 52.19 (s, CB-BA), 28.27 (s, CA-BA, CA-G), 21.06 (s, C-Bn-imi), 14.20 (s, (CH*_*3*_*)*_*3*_*-Boc).*

MS: *m/z* calculated for C_21_H_28_N_4_O_5_ 417.47, found 417.2 [M+H]^+^.

#### Boc-His(Bn)-Ala-OMe

Yield: 48.38%.

^1^H NMR (500 MHz, CDCl_3_) δ ppm : 7.47 (s, 1H, CH), 7.39–7.10 (m, 5H, Ph), 6.72 (s, 1H, CH), 6.12 (s, 1H, NH), 5.04 (s, 2H, CH_2_), 4.33 (m, 1H, CH), 3.66 (s, 3H, COOCH_3_), 3.44 (m, 2H, CH_2_), 2.90 (m, 2H, CH_2_), 2.42 (m, 2H, CH_2_), 1.41 (s, 9H, CH_3_-Boc).

^13^C NMR (101 MHz, DMSO-*d*_*6*_) δ_C_:172.48 (C16), 171.91 (C27), 155.67 (C18), 138.02 (C4), 136.58 (C10), 135.71 (C12), 129.02 (C8), 128.39 (C2,C6), 127.40 (C3, C6), 117.36 (C1), 79.80 (C1), 54.77 (C14), 51.69 (C31), 50.98 (C7), 34.80 (C26), 34.65 (C28), 30.58 (C13), 28.26 (C22, C23, C24).

MS: *m/z* calculated for C_22_H_30_N_4_O_5_ 431.50, found 431.2 [M+H]^+^.

#### Boc-His(Bn)-Leu-OMe

Yield: 47.59%.

^1^H NMR (500 MHz, CDCl_3_) δ ppm: 7.48 (s, 1H, CH), 7.41–7.10 (m, 5H, Ph), 6.75 (s, 1H, CH), 6.16 (d, *J* = 5.7 Hz, 1H, NH), 5.04 (m, 2H, CH_2_), 4.52 (m, 1H, CH), 4.43 (m, 1H, CH), 3.68 (s, 3H, COOCH_3_), 2.97 (m, 2H, CH_2_), 1.54 (m, 3H, CH_2_, CH), 1.43 (s, 9H, CH_3_-Boc), 0.93 (d, *J* = 5.4 Hz, CH_3_).

^13^C NMR (101 MHz, DMSO-*d*_*6*_) δ_C_: 173.08 (C27), 171.68 (C16), 155.71 (C18), 138.25 (C4), 136.44 (C10), 135.66 (C12), 129.02 (C2, C6), 128.39 (C3, C5), 127.45 (C1), 117.40 (C8), 79.78 (C21), 52.11 (C14), 50.98 (C34), 50.78 (C26), 50.48 (C7), 41.14 (C28), 30.53 (C13), 28.27 (C22, C23, C24), 24.63 (C31), 22.86 (C32), 22.77 (C33).

MS: *m/z* calculated for C_25_H_36_N_4_O_5_ 473.58, found 473.3 [M+H]^+^.

#### Boc-His(Bn)-Ile-OMe

Yield: 47.93%.

^1^H NMR (500 MHz, CDCl_3_) δ ppm: 7.70 (s, 1H, CH), 7.47–7.14 (m, 5H, Ph), 6.79 (s, 1H, CH), 6.30 (d, *J* = 5.2 Hz, 1H, NH), 5.09 (m, 2H, CH_2_), 4.48 (m, 2H, CH), 3.70 (s, 3H, COOCH_3_), 3.07 (m, 2H, CH_2_), 1.86 (m, 1H,CH), 1.44 (s, 9H, CH_3_-Boc), 1.15 (m, 5H, CH_2_, CH_3_), 0.82 (d, *J* = 6.9 Hz, CH_3_).

^13^C NMR (101 MHz, DMSO-*d*_*6*_) δ_C_: 172.03 (C27), 171.39 (C16), 155–160 (C18), 135.81 (C4), 135.78 (C10), 134.98 (C12), 129.18 (C2, C6), 128.71 (C3, C5), 127.74 (C1), 117.54 (C8), 81.87 (C21), 56.74 (C26), 51.92 (C14), 51.42 (C30), 37.54 (C7), 30,18 (C31), 28.31 (C22, C23, C24), 25.04 (C33), 15.41 (C32), 11.58 (C34).

MS: *m/z* calculated for C_25_H_36_N_4_O_5_ 473.58, found 473.3 [M+H]^+^.

#### Boc-His(Bn)-Phe-OMe

Yield: 37.17%.

^1^H NMR (500 MHz, CDCl_3_) δ ppm: 7.35 (s, 1H, NH), 7.43–6.90 (m, 10H, Ph), 6.75 (s, 1H, CH), 6.27 (s, 1H, NH), 5.03 (s, 2H, CH_2_), 4.79 (m, 1H, CH), 4.43 (m, 1H, CH), 3.64 (s, 3H, COOCH_3_), 2.98 (m, 4H, CH_2_), 1.42 (s, 9H, CH_3_-Boc).

^13^C NMR (101 MHz, DMSO-*d*_*6*_) δ_C_:171.53 (C27), 171.32 (C16), 155.67 (C18), 138.18 (C30), 136.36 (C4), 136.09 (C10), 135.49 (C12), 129.24 (C8), 129.04 (C3, C5, C31, C35), 127.55 (C2,C6, C32, C34), 117.51 (C1), 117.34 (C33), 79.35 (C21), 54.48 (C26), 54.07 (C14), 53.41 (C37), 52.10 (C7), 38.05 (C18), 30.16 (C13), 28.28 (C22, C23, C24).

MS: *m/z* calculated for C_28_H_34_N_4_O_5_ 507.59, found 507.2 [M+H]^+^.

#### Boc-His(Bn)-D-Glu-(OMe)_2_

Yield: 50.37%.

^1^H NMR (500 MHz, CDCl_3_) δ ppm: 7.70 (s, 1H, CH), 7.25 (m, 5H, Ph), 6.73 (s, 1H, CH), 6.10 (s, 1H, NH), 5.05 (m, 2H, CH_2_), 4.53 (m, 1H, CH), 3.81 (m, 1H, CH), 3.59 (s, 6H, COOCH_3_), 2.90 (m, 2H, CH_2_), 2.17 (m, 4H, CH_2_), 1.34 (s, 9H, CH_3_-Boc).

^13^C NMR (101 MHz, DMSO-*d*_*6*_) δ_C_: 173.40 (C32), 173.30 (C27), 171.14 (C16), 156.87 (C18), 137.95 (C4), 136.45 (C10), 135.59 (C12), 129.02 (C2, C6), 128.42 (C3, C5), 127.52 (C1), 117.43 (C8), 77.37 (C21), 52.36 (C14), 51.79 (C26), 51.69 (C36). 51.43 (C35), 51.02 (C7), 30.05 (C31), 29.69 (C13), 28.26 (C22, C23, C24), 27.33 (C28).

MS: *m/z* calculated for C_25_H_34_N_4_O_7_ 503.56, found 503.2 [M+H]^+^.

#### Boc-His(Bn)-Asp-(OMe)_2_

Yield: 42.79%.

^1^H NMR (500 MHz, CDCl_3_) δ ppm: 7.69 (s, 1H, CH), 7.56–7.33 (m, 5H, Ph), 6.63 (s, 1H, CH), 6.23 (s, 1H, NH), 5.05 (m, 2H, CH_2_), 4.80 (m, 1H, CH), 4.43 (m, 1H, CH), 3.71 (s, 3H, COOCH_3_), 3.68 (s, 3H, COOCH_3_), 2.93 (m, 2H, CH_2_), 2.70 (m, 2H, CH_2_), 1.45 (s, 9H, CH_3_-Boc).

^13^C NMR (101 MHz, DMSO-*d*_*6*_) δ_C_:171.63 (C10, C15), 170.93 (C32), 155.56 (C7), 138.19 (C20), 136.58 (C26), 135.76 (C28), 129.02 (C18, C22), 128.39 (C19, C21), 127.47 (C17), 117.25 (C24), 81.77 (C2), 54.52 (C30), 52.63 (C12), 51.94 (C35), 50.95 (C23), 48.60 (C9), 36.25 (C14), 30.28 (C29), 28.31 (C3, C4, C5).

MS: *m/z* calculated for C_24_H_32_N_4_O_7_ 489.53, found 489.1 [M+H]^+^.

#### Boc-His(Bn)-Arg(NO_2_)-OMe

Yield: 45.19%.

^1^H NMR (500 MHz, CDCl_3_) δ ppm: 7.70 (s, 1H, CH), 7.51–7.18 (m, 5H, Ph), 6.75 (s, 1H, CH), 5.03 (m, 2H, CH_2_), 4.54 (m, 2H, CH), 3.74 (s, 3H, COOCH_3_), 3.51 (m, 2H, CH_2_), 3.14 (m, 2H, CH_2_), 1.83 (m, 2H, CH_2_), 1.57 (m, 1H, CH), 1.46 (s, 9H, CH_3_-Boc), 1.26 (m, 1H, CH), 0.09 (s, 1H, NH).

^13^C NMR (101 MHz, DMSO-*d*_*6*_) δ_C_:171.95 (C27), 171 (C16), 159.37 (C33), 155.77 (C18), 136.56 (C15), 135.46 (C12), 128.08 (C2, C6), 128.53 (C3, C5), 127.67 (C1), 117.51 (C8), 77.36 (C21), 54.64 (C26), 53.45 (C14), 52.62 (C41), 50.75 (C7), 40.28 (C31), 29.70 (C29), 29.55 (C13), 28.29 (C22, C23, C24), 24.05 (C30).

MS: *m/z* calculated for C_25_H_36_N_8_O_7_ 561.60, found 561.3 [M+H]^+^.

### Unprotected dipeptidomimetics

*General procedure*: Removal of a a *tert*-butyloxycarbonyl (Boc) protective group was carried with TFA (trifluoroacetic acid). The mixture of dipeptidomimetics in TFA (in a proportion of 1 g to 5 ml, respectively) was stirred for about 2 h in room temperature and therefore washed several times with anhydrous diethyl ether, to ensure complete separation of TFA.

TFAxβ-Ala-Gly-OMe (TFAxBAG) [C_6_H_12_N_2_O_3_^+^]**:** *m/z* calculated: 161.18, found: *m/z* [M+H]^+^: 161.1;

TFAxβ-Ala-Ala-OMe (TFAxBAA) [C_7_H_14_N_2_O_3_^+^]: *m/z* calculated: 175.21, found: *m/z* [M+H]^+^: 175.1;

TFAxβ-Ala-Val-OMe (TFAxBAV) [C_9_H_18_N_2_O_3_^+^]: *m/z* calculated: 402.26, found [2M-H]^-^: 402.8;

TFAxβ-Ala-Leu-OMe (TFAxBAL) [C_10_H_20_N_2_O_3_^+^]: *m/z* calculated: 217.28, found [M+H]^+^: 217.6 ;

TFAxβ-Ala-Ile-OMe (TFAxBAI) [C_10_H_20_N_2_O_3_^+^]: m/z calculated: 217.28, found [M+H]^+^: 217.2;

TFAxβ-Ala-Phe-OMe (TFAxBAF) [C_13_H_18_N_2_O_3_^+^]: m/z calculated: 249.30, found [M-H]^-^: 248.9;

TFAxβ-Ala-Glu-(OMe)_2_ (TFAxBAE) [C_10_H_18_N_2_O_5_^+^]: m/z calculated: 247.27, found [M+H]^+^: 247.1;

TFAxβ-Ala-Asp-(OMe)_2_ (TFAxBAD) [C_9_H_17_N_2_O_5_^+^]: m/z calculated: 233.24, found [M+H]^+^_:_ 233.4;

TFAxβ-Ala-Arg(NO_2_)-OMe (TFAxBAR) [C_10_H_20_N_6_O_5_^+^]: m/z calculated: 305.16, found [M+H]^+^_:_ 305.2;

TFAxHis(Bzl)-Gly-OMe (TFAxHG) [C_16_H_20_N_4_O_3_^+^]: m/z calculated: 317.15, found [M+H]^+^_:_ 317.2;

TFAxHis(Bzl)-Ala-OMe (TFAxHA) [C_17_H_22_N_4_O_3_^+^]: m/z calculated: 331.18, found [M+H]^+^: 331.2;

TFAxHis(Bzl)-Val-OMe (TFAxHV) [C_19_H_26_N_4_O_3_^+^]: m/z calculated: 359.44, found [M+H]^+^_:_ 359.5;

TFAxHis(Bzl)-Leu-OMe (TFAxHL) [C_20_H_28_N_4_O_3_+]: m/z calculated: 405.22, found [M+CH_3_OH+H]^+^: 405.2;

TFAxHis(Bzl)-Ile-OMe (TFAxHI) [C_20_H_28_N_4_O_3_^+^]: m/z calculated: 373.47, found [M+H]^+^_:_ 373.2;

TFAxHis(Bzl)-Phe-OMe (TFAxHF) [C_23_H_26_N_4_O_3_^+^]: m/z calculated: 407.48, found [M+H]^+^_:_ 407.1;

TFAxHis(Bzl)-Glu-(OMe)_2_ (TFAxHE) [C_20_H_26_N_4_O_5_^+^]: m/z calculated: 444.45, found [M+ACN+H]^+^: 445.3;

TFAxHis(Bzl)-Asp-(OMe)_2_ (TFAxHD) [C_19_H_24_N_4_O_5_^+^]: m/z calculated: 386.43, found [M-2H]^-^_:_ 385.2

TFAxHis(Bzl)-Arg(NO_2_)-OMe (TFAxHR) [C_20_H_28_N_8_O_5_^+^]: m/z calculated: 461.49, found [M+H]^+^: 461.2.

### Altered dipeptidomimetics

Two chosen histidine mimetics **Boc-HV** and **Boc-HR**, underwent dehydrogenation in order to deprotect the imidazole polar nitrogen, according to standard procedure:

Boc-His(Bn)-Val-OMe / Boc-His(Bn)-Arg(NO_2_)-OMe (0.29 mmol) was dissolved in methanol (10 ml), and then allowed to cool down in an ice bath until 0 °C. Pd/C (12 mg) was then added to the reaction mixture and subjected to hydrogenation for 18 h. The reaction mixture was then filtered on a Celite bed, and solvent was evaporated. Resulting residue was purified by preparative TLC using CHCl_3_:MeOH (9:1) due to small quantities of the product. Immediately, deprotection of Boc was performed and desired products, **dHV** and **dHR** were both obtained in 96% yield.

TFAxHis-Val-OMe** (dHV)**: *m/z* calculated for C_12_H_21_N_4_O_3_^+^: 269.16, found [M+H]^+^: 269.6

TFAxHis(Bzl)-Arg(NO_2_)-OMe **(dHR)**: *m/z* calculated for C_13_H_23_N_8_O_5_^+^: 353.18, found [M-H_2_O-H]^-^: 353.7

### Biological studies

MTT assay was used to evaluate the effect of proposed compounds upon cell proliferation of MCF-7 and HDFa human cell lines. MCF-7 is a breast cancer cell line with the highest amount of available scientific data and is characterized as having non-invasive profile and poorly-aggressive^114^. HDFa is Human Dermal Fibroblast cell line, established as good negative control for anti-cancer drug testing^63,67^. Both MCF-7 and HDFa cell lines were acquired from the American Type Culture Collection (ATCC; Manassas, VA, USA) and cultured in PAN Biotech DMEM supplemented with 10% fetal bovine serum (FBS), 6 μg/mL of penicillin-G, and 10 μg/mL of streptomycin.

### MTT procedure

Cells were seeded into 96-well plates, at a density of 0.8 × 10^5^ cells/ml, in DMEM supplemented with 10% FBS and 1% P/S (antibiotics). Cells were incubated at 37 °C in 5% CO_2_ for 24 h. Then the medium was exchanged for serum-free DMEM with the appropriate concentrations of analyzed peptides. After 48 h of later incubation the cells in each well were seeded with 11 µl of fresh MTT ((3-(4,5-dimethyl-thiazol-2-yl)-2,5-diphenyl-tetrazolium bromide) to receive a terminal concentration of 0.5 mg/ml. Such prepared plates were incubated in 5% CO_2_ at 37 °C for 2 h. Culture medium was then removed carefully and the remaining formazan crystals were dissolved in 100 µl DMSO. The absorbance at 570 nm and 660 nm was measured using microplate reader. The cell viability percentage was calculated based on the absorbance ratio between cell culture treated with tested compounds and the untreated control multiplied by 100 represents cell viability (percentage of control, %).

### Detection of apoptosis

Apoptotic and necrotic cells were detected by Annexin V binding and propidium iodide (PI) uptake using apoptosis assay kit (BD Pharmingen, USA). MCF-7 cells were seeded on a 6-well plate. After 24h, the cells were treated with 2 different concentrations of tested ligand **dHV**. The next day the cells were collected, washed twice with PBS (NaCl 0.138 M; KCl 0.0027 M; pH 7.4) and resuspended in binding buffer (50 mM HEPES: 4-(2-hydroxyethyl)-1-piperazineethanesulfonic acid, 700 mM NaCl, 12.5 mM CaCl_2_, pH 7.4). 5 μL of Annexin V and 5 μL propidium iodine were added to the cells, gently shaken and hidden in the dark, at the room temperature for 15 min. Then cells were diluted in a buffer and assessed using BD FACSArray (BD Biosciences, San Jose, CA, USA). Plots from the gated cells illustrated the populations of viable (Annexin V− PI−) cells, apoptotic (Annexin V+ PI−), apoptotic/necroptotic (Annexin V+ PI+) cells and dead (Annexin V− PI+) cells.

### Statistical analysis

Statistical analysis was prepared in GraphPrism 5 software. Data showing the percentage of viable cells are presented as the mean ± standard error. Statistical analysis was determined by one-way analysis of variance (ANOVA) and Tukey’s posthoc test by comparing obtained values to control. The null hypothesis here was that the differences observed for the cells viability in the independent series of experiments should not be due to random chance, so the difference between the means observed for tested entities are bigger than those shown for the control.

### Computational studies

#### Target fishing

Briefly speaking, target fishing is an in silico method for the theoretical prediction of many biological targets for one chosen molecule. There are two methods for pursuing target fishing: ligand- and receptor-based, out of which we used the latter.

Pharmacophore-based server, PharmMapper (http://www.lilab-ecust.cn/pharmmapper/)^115–117^ lets its users search through different pharmacophore models of 23 thousand proteins to find the best fitting, for a ligand of interest. We used this tool to generate lists of 400 targets from the set “Druggable Pharmacophore Models (v2017, 16159)”. The results were then be cut by the minimum value of Fit > 2.5.

In order to assess which genes from the obtained lists are present in humans and to switch identifier type from PDB to UniProtKB, we used the “Retrieve/ID mapping function” in UniProt Database (https://www.uniprot.org/). Then, the identifiers were downloaded and given for the right PDB ID in PharmMapper list and then compared with the list of cancer-relevant proteins. For the creation of such list we used the GeneCards database^118^ (https://www.genecards.org), which is a repository for human genomes, transcriptomes and proteomes. By simply using a word “cancer” as a keyword, we obtained 25.965 results of genes involved in cancerogenesis. The duplicates were deleted and only the results with the minimum relevance of 5.0 were taken into later consideration. This led us to the assessment of 2259 enzymes related to cancer, which we saved as our “Cancer Database”.

To fully understand the relationship between a substance effected targets, pathways and diseases, Cytoscape software was used to build a network diagram.

#### Protein–protein interaction (PPI) network

Intersection genes for sets with native dipeptides, **BAX** and **HX**, were put separately into the STRING Database (Search Tool for the Retrieval of Interacting Genes; https://string-db.org/) in order to find possible existence of interactions between the given set of proteins, including both direct and indirect connections. Results are presented with a given measure of confidence, ranging from 0 to 1. The highest possible threshold was chosen (confidence value of at least 0.9), FDR stringency 5% and species option set to “Homo sapiens”.

The Compound-Target (CT) Network was prepared with the use of Cytoscape (https://cytoscape.org/), which is an open-source software enabling the visualization and analysis of molecular interaction networks^119^. The results from STRING were implemented into Cytoscape and with the use of “Network Analyzer” plug-in—the topology parameters were calculated.

We set the minimum of connections of a given node to 10, meaning this is the minimal degree, taken into PPI network.

#### Molecular docking

The X-ray structures of the Caspase-3 and Rac1 used for molecular modeling studies were taken from the Protein Databank (Accession Codes, respectively: 1GFW and 1MH1). The docking analysis was carried out after standard preparation procedures in AutoDock Vina 1.1.2 software (The Molecular Graphic Laboratory, The Scripps Research Institute, La Jolla, CA, USA)^120^, including removal of co-crystalized ligands and water molecules, building a model with missing atoms, addition of hydrogen atoms and Gasteiger charges to each atom.

For caspase-3, a grid box size of 25 × 25 × 25 Å, was centered on the Cα of Cys163 (x = 34.773, y = 32.635, z = 35.367). With Rac1, we decided to set the center of the grid box on the carbon alpha (x = 14.068, y = 37.085, z = 13.240) of Trp56, which with literature search we found as the most relevant to the effectivity of binding of Rac1 with guanidine nucleotide exchange factors. This means that if a given entity binds Trp56, it is therefore possibly able to inhibit Rac1 function^121–123^. After validation, using the inhibitor of Rac1 with the already established position in Rac1 active site^123^ we were able to set the grid box to 54 × 54 × 46 Å. We also compared our hypothesized grid box size with the dockings available in the literature and we found similar values, which led us to believe our settings are done with care^123^.

#### Enrichment analysis (GO and KEGG)

The database for annotation, visualization and integrated discovery (DAVID: https://david.ncifcrf.gov/) is a set for a better understanding of biological functions of a given substance, based on its list of potential gene targets. Gene ontology (GO) function and Kyoto encyclopedia of genes and genomes (KEGG) pathway enrichment analyses were performed, in order to analyze dominant activities and signaling pathways in which it our substances of interest can participate. KEGG is a manually governed database created by Minoru Kanehisa in 1995. Today it’s large-scale coverage allows for genomic identification, comparison and functionality assessment of many taxonomic trees^83,124,125^. KEGG’s immense database size allowed us to analyzes enzymatic input as a functional network, estimating the probability of possible disruption of specific organellum, pathway or disease. KEGG sufficiently interprets large data sets and categorizes them into possible functionalities^126–129^ and is successfully embraced in medicine, for example in cancer, cardiac, muscle, neurodegenerative and gastrointestinal research^130–138^.

In order to make our set more statistically significant, only categories with *p* < 0.05 (-log_10_(p) > 1.3) were taken into consideration, with of FDR (False Discovery Rate) multiple testing correction, which reflects that the proportion of false positives in a given set of data cannot exceed proposed value. If FDR < 0.05 then the number of false positives in our data does not exceed 5% of all positive tests.

#### ADME

ADME, as well as BOILED-Egg calculation and depiction—were all performed using Python, with modules: RDKit, matplotlib and shapely.

## Supplementary Information

Below is the link to the electronic supplementary material.

Supplementary Material 1

## Data Availability

All data generated or analysed during this study are included in this published article (and its Supplementary Information files), available via the publisher’s website.
