# Supplementary material for: Potential medical applicability of N-β-Ala and N-His dipeptidomimetics against breast cancer: short in vitro and in silico screening
Source: Sci Rep. 2026 May 16;16:22232. doi: 10.1038/s41598-026-50964-7 (PMC13369175; doi:10.1038/s41598-026-50964-7)
Supplement: Supplementary file 1 — Supplementary Material 1 [file 41598_2026_50964_MOESM1_ESM.pdf]

Potential medical applicability of *N*-β-Ala and *N*-His dipeptidomimetics against breast cancer – short *in vitro* & *in silico* screening

Klaudia Chmielewska[1,4]‡, Justyna Budka[2], Katarzyna Kozłowska-Tylingo[3], Iwona Inkielewicz-Stepniak[2], Krystyna Dzierzbicka[1]‡

[1]*Department of Organic Chemistry, Faculty of Chemistry, Gdansk University of Technology, G. Narutowicza 11/12, PL-80-233 Gdansk, Poland*

[2]*Department of Pharmaceutical Pathophysiology, Medical University of Gdansk, Debinki 7, PL-80-211 Gdansk, Poland*

[3]*Department of Pharmaceutical Technology and Biochemistry, Gdansk University of Technology, G. Narutowicza 11/12, PL-80-233 Gdansk, Poland*

[4]*Laboratory of Environmental Chemometrics, Faculty of Chemistry, University of Gdansk, Wita Stwosza 63, 80-308 Gdansk, Poland*

‡ corresponding authors: klaudia.chm@gmail.com (Klaudia Chmielewska), krydzier@pg.edu.pl (Krystyna Dzierzbicka)

1. COMPUTATIONAL STUDIES

1.1. Target Fishing

Table 1. Targets related to cancerogenesis found for **BAX** and **HX** sets with PharmMapper.

Set identifier	Targets
**BAX**	O00299, O00444, O14776, O60341, O60381, O60603, O75369, O75533, P00450, P00491, P00492, P01579, P01889, P01911, P02751, P02766, P04275, P04278, P04439, P04626, P05106, P06213, P06396, P06756, P07477, P08069, P08253, P10276, P10646, P10747, P10826, P11387, P11388, P11802, P11836, P11926, P12004, P12272, P12429, P12956, P13010, P13569, P13591, P13631, P15692, P16444, P18054, P18858, P18887, P19438, P19793, P21266, P22301, P23921, P24385, P24864, P24941, P25311, P26358, P27487, P29965, P30153, P35240, P35625, P35658, P35659, P35680, P35790, P40225, P42575, P43246, P43355, P45984, P46013, P46527, P46531, P46940, P49711, P50613, P51513, P51532, P51946, P52701, P54727, P54756, P54760, P60953, P61024, P61769, P62877, P63000, P63151, P68431, P78536, P98170, Q01543, Q03518, Q04721, Q04724,

	Q06455, Q12884, Q13309, Q13873, Q15375, Q15399, Q15788, Q15797, Q16853, Q53EL6, Q92889, Q969H0, Q96A58, Q96QZ7, Q99075, Q99707, Q9BUZ4, Q9GZV9, Q9HB71, Q9NTG7, Q9NTK5, Q9UBN7, Q9UI42, Q9UPW6
**HX**	P17693, P01920, P45984, P15692, Q30201, P24941, P10747, P30153, Q9UBN7, Q15306, P01909, P07900, P09211, P11802, Q13309, P18054, P08069, Q15399, P62877, P53041, Q13263, O60341, P35222, Q9Y297, P06746, O60315, P52789, P12272, Q9NTX7, P52701, O60603, P46527, P61024, P13010, P13569, P22301, P02751, P10721, P60953, P12956, P61769, P01579, O75369, P03956, Q9NTK5, P49862, Q9UM73, Q00535, P04275, P50613, P24864, P02766, P43358, P63151, P24385, P21980, Q04637, P51532, P11836, Q96QZ7, P29320, P23921, P02768, P35240, Q9UIF7, Q9UPW6, P26358, Q9BUZ4, P63000, P46013, Q16853, P43246, P51946, P05106, P35968, P35680, P37231, Q15303, Q99707, Q9Y2U5, Q9UQ80, P07288, Q9NZC7, P68431, P08134, Q01543, P08253, Q969H0, P18440, P40225, P55055, P04439, P43355, Q12923, Q02548, P49023, O00330, O60583, P05181, Q15047, P02671, Q99075, P35243, P10828, P25311, O00299, P04626, P78358, Q13029, P19793, Q15788, Q13489, P05164, Q13541, P00450, Q05655, P01889, Q96A58, P27487, P54727, Q9Y6Q9, P29508, P42679, P01911, P35658, Q13257, O00444, P11388, P04637, Q9Y617, O15075, P78536, P05019, P08833, P22692, Q9HB71, Q00534, Q93034

1.2. PPI Network

Table 2. Basic data on the uncleaned and cleaned (after Degree>9 cut-off).

	BAX	HX
Number of nodes	123	138
Number of nodes after cut-off	26	18
Number of edges	295	275
Clustering coefficient	0.377	0.376

Table 3. Summary of obtained network data for the sets **.** The 12 targets that create the nodes for both of the sets are underlined.

No.	BAX	Degree	Betweenness	Closeness	HX	Degree	Betweenness	Closeness
1	CCND1	17	0.1509	0.4008	TP53	24	0.3023	0.4348
2	PCNA	16	0.0578	0.3414	HSP90AA1	18	0.1906	0.4237
3	VEGFA	14	0.0934	0.3929	CTNNB1	17	0.1753	0.4098
4	CDK2	14	0.0433	0.3561	CDKN1B	16	0.0896	0.4065

5	FN1	14	0.1239	0.3613	ITGB3	14	0.0937	0.3846
6	XRCC6	14	0.0536	0.3173	CCNE1	13	0.0228	0.3509
7	CDKN1B	13	0.1293	0.3779	VEGFA	13	0.0479	0.3774
8	NOTCH1	13	0.1948	0.4008	IGF1	13	0.0769	0.3676
9	CCNE1	12	0.0066	0.3438	CDK2	12	0.0092	0.3484
10	CKS1B	12	0.0255	0.3486	RAC1	12	0.0342	0.3876
11	CCNH	12	0.0534	0.3438	CDK4	12	0.0205	0.3717
12	MSH2	11	0.0109	0.2964	PXN	11	0.0105	0.3484
13	CD28	11	0.1412	0.3037	FN1	11	0.0410	0.3497
14	RAC1	11	0.0530	0.3511	CDC42	11	0.0307	0.3676
15	RAD23B	11	0.0140	0.3046	ERBB2	11	0.1000	0.3831
16	XRCC5	11	0.0064	0.2895	CDK5	11	0.0304	0.3559
17	CDK4	11	0.0116	0.3426	CCNH	10	0.0197	0.3448
18	CDK7	11	0.0398	0.3426	CCND1	10	0.0132	0.3636
19	ITGAV	10	0.0641	0.3626				
20	ERCC4	10	0.0099	0.3037				
21	CDC42	10	0.0411	0.3278				
22	ERBB2	10	0.0663	0.3680				
23	TOP2A	10	0.0757	0.3402				
24	MSH6	10	0.0015	0.2886				
25	ITGB3	10	0.0773	0.3613				
26	LIG1	10	0.0031	0.2886				

1.3. Gene Ontology and KEGG

Table 4. Gene Ontology results, after –log(p) or FDR > 1.3 cut-off, for the set **BAX**.

	**Term**	**-log(p)**	**-log(FDR)**
BP	G1/S transition of mitotic cell cycle	9.96	7.20
	cell division	6.25	3.79
	vascular endothelial growth factor receptor signaling pathway	5.44	3.23
	transcription-coupled nucleotide-excision repair	5.39	3.23
	positive regulation of cyclin-dependent protein serine/threonine kinase activity	5.24	3.18
	mismatch repair	4.73	2.78
	telomere maintenance	4.70	2.78
	substrate adhesion-dependent cell spreading	4.62	2.77
	positive regulation of protein phosphorylation	4.46	2.65
	positive regulation of cell proliferation	4.30	2.54
	cell proliferation	3.75	2.03
	DNA repair	3.43	1.74
	protein phosphorylation	3.31	1.66
	positive regulation of gene expression	3.25	1.63

	positive regulation of viral genome replication	3.17	1.58
	nucleotide-excision repair, preincision complex assembly	3.07	1.54
	positive regulation of transcription, DNA-templated	3.07	1.54
	positive regulation of cell cycle	2.94	1.45
	regulation of protein kinase activity	2.94	1.45
CC	cyclin-dependent protein kinase holoenzyme complex	8.43	6.67
	nucleoplasm	8.39	6.67
	nuclear chromosome, telomeric region	4.56	3.01
	nucleus	4.07	2.65
	cyclin E1-CDK2 complex	2.56	1.38
	MutSalpha complex	2.56	1.38
	integrin alphav-beta3 complex	2.56	1.38
MF	damaged DNA binding	9.45	7.45
	cyclin-dependent protein serine/threonine kinase regulator activity	8.30	6.59
	protein C-terminus binding	6.67	5.14
	kinase activity	5.95	4.55
	protein binding	5.72	4.41
	enzyme binding	5.14	3.91
	protein kinase binding	4.84	3.68
	MutLalpha complex binding	4.50	3.40
	ATP binding	3.74	2.69
	double-stranded DNA binding	3.64	2.64
	protease binding	3.36	2.40
	identical protein binding	3.19	2.32
	extracellular matrix binding	3.17	2.32
	fibronectin binding	3.17	2.32
	coreceptor activity	3.05	2.22
	DNA-dependent ATPase activity	2.99	2.19
	cyclin-dependent protein serine/threonine kinase activity	2.94	2.17
	protein kinase activity	2.75	2.00
	single thymine insertion binding	2.53	1.80
	single guanine insertion binding	2.35	1.67
	dinucleotide insertion or deletion binding	2.35	1.67
	guanine/thymine mispair binding	2.23	1.59
	Rho GDP-dissociation inhibitor binding	2.23	1.59
	oxidized purine DNA binding	2.13	1.57
	C-X3-C chemokine binding	2.13	1.57
	macromolecular complex binding	2.13	1.57
	5'-deoxyribose-5-phosphate lyase activity	2.13	1.57
	neuregulin binding	2.13	1.57
	single-stranded DNA binding	2.08	1.54
	vascular endothelial growth factor receptor 2 binding	2.05	1.53
	histone deacetylase binding	2.00	1.49
	double-stranded telomeric DNA binding	1.93	1.43

Table 5. Gene Ontology results, after –log(p) or FDR > 1.3 cut-off, for the set **HX**.

	**Term**	**-log(p)**	**-log(FDR)**
BP	vascular endothelial growth factor receptor signaling pathway	8.13	5.32
	G1/S transition of mitotic cell cycle	7.36	4.86
	cell-matrix adhesion	5.76	3.43
	positive regulation of protein phosphorylation	5.16	2.96
	positive regulation of neuron apoptotic process	4.98	2.88
	cell division	4.72	2.69
	mitotic G1 DNA damage checkpoint	4.28	2.32
	positive regulation of peptidyl-tyrosine phosphorylation	4.14	2.27
	positive regulation of cell proliferation	4.12	2.27
	positive regulation of gene expression	3.93	2.13
	platelet degranulation	3.84	2.08
	response to drug	3.68	1.96
	positive regulation of cyclin-dependent protein serine/threonine kinase activity	3.58	1.89
	cell proliferation	3.37	1.72
	regulation of protein kinase activity	3.27	1.68
	positive regulation of cell cycle	3.27	1.68
	substrate adhesion-dependent cell spreading	3.18	1.61
	positive regulation of DNA replication	3.09	1.55
	cellular protein localization	3.07	1.55
	protein phosphorylation	3.02	1.53
	cell adhesion	3.01	1.53
	positive regulation of fibroblast proliferation	2.87	1.41
	positive regulation of transcription, DNA-templated	2.82	1.38
	positive regulation of epithelial cell proliferation	2.78	1.36
	DNA damage response, signal transduction by p53 class mediator resulting in cell cycle arrest	2.75	1.35
	response to wounding	2.74	1.35
CC	cyclin-dependent protein kinase holoenzyme complex	6.52	4.44
	cytosol	4.53	2.75
	nucleoplasm	4.33	2.73
	focal adhesion	3.40	2.01
	lamellipodium	3.38	2.01
	nucleus	3.23	1.93
	platelet alpha granule lumen	2.93	1.70
	cyclin E1-CDK2 complex	2.73	1.56
	cytoplasm	2.67	1.55
	membrane	2.62	1.55
	ruffle membrane	2.58	1.55
	alphav-beta3 integrin-IGF-1-IGF1R complex	2.43	1.45
	melanosome	2.41	1.45
	bicellular tight junction	2.31	1.38
	protein complex	2.21	1.31
	protein kinase binding	7.38	5.33

MF	cyclin-dependent protein serine/threonine kinase regulator activity	6.42	4.81
	protein phosphatase binding	6.39	4.81
	kinase activity	5.51	4.06
	Rho GDP-dissociation inhibitor binding	5.24	3.88
	protein binding	4.83	3.55
	identical protein binding	4.22	3.00
	enzyme binding	3.54	2.39
	protein kinase activity	3.42	2.31
	cyclin-dependent protein serine/threonine kinase activity	3.28	2.22
	ErbB-3 class receptor binding	2.40	1.40
	protease binding	2.34	1.40
	histone deacetylase binding	2.33	1.40
	integrin binding	2.31	1.40
	vascular endothelial growth factor receptor 2 binding	2.22	1.34

Table 6 KEGG results after –log(p) or FDR > 1.3 cut-off, for the set **BAX**.

**Term**	**-log(p)**	**-log(FDR)**
Pathways in cancer	10.10	8.22
Small cell lung cancer	8.00	6.43
Cell cycle	6.85	5.46
Nucleotide excision repair	6.34	5.06
PI3K-Akt signaling pathway	5.92	4.74
Pancreatic cancer	5.62	4.53
Proteoglycans in cancer	5.44	4.41
Focal adhesion	5.36	4.39
Viral carcinogenesis	4.25	3.33
Mismatch repair	4.20	3.32
Measles	4.10	3.27
Hepatitis B	3.92	3.13
Prostate cancer	3.66	2.90
MicroRNAs in cancer	3.46	2.74
Bladder cancer	3.44	2.74
Colorectal cancer	2.91	2.24
p53 signaling pathway	2.81	2.17
Regulation of actin cytoskeleton	2.26	1.64
Thyroid hormone signaling pathway	2.15	1.55
Non-small cell lung cancer	1.80	1.23
Viral myocarditis	1.78	1.23
VEGF signaling pathway	1.73	1.19
Renal cell carcinoma	1.66	1.15
Adherens junction	1.60	1.11
Chronic myeloid leukemia	1.59	1.11
Bacterial invasion of epithelial cells	1.53	1.07

Rap1 signaling pathway	1.45	1.01
ECM-receptor interaction	1.44	1.01
HIF-1 signaling pathway	1.36	0.95
Non-homologous end-joining	1.35	0.95
T cell receptor signaling pathway	1.33	0.94
FoxO signaling pathway	1.10	0.73
Phagosome	1.02	0.66

Table 7 KEGG results after –log(p) or FDR > 1.3 cut-off, for the set **HX**.

**Term**	**-log(p)**	**-log(FDR)**
Pathways in cancer	12.96	11.13
Proteoglycans in cancer	11.25	9.72
Prostate cancer	10.94	9.59
PI3K-Akt signaling pathway	10.39	9.17
Focal adhesion	9.49	8.37
Pancreatic cancer	8.19	7.14
Viral carcinogenesis	7.98	7.00
Small cell lung cancer	7.48	6.56
Cell cycle	6.50	5.62
p53 signaling pathway	6.37	5.54
Bladder cancer	5.61	4.83
Measles	4.89	4.14
Hepatitis B	4.71	4.00
Bacterial invasion of epithelial cells	4.49	3.81
MicroRNAs in cancer	4.39	3.74
Rap1 signaling pathway	3.94	3.31
Endometrial cancer	3.59	2.99
Non-small cell lung cancer	3.49	2.92
VEGF signaling pathway	3.38	2.84
Colorectal cancer	3.36	2.84
Glioma	3.30	2.80
Adherens junction	3.19	2.73
Melanoma	3.19	2.73
Chronic myeloid leukemia	3.17	2.73
Regulation of actin cytoskeleton	2.83	2.40
HIF-1 signaling pathway	2.81	2.40
Thyroid cancer	2.65	2.25
Leukocyte transendothelial migration	2.58	2.22
Thyroid hormone signaling pathway	2.58	2.22
FoxO signaling pathway	2.40	2.05
Wnt signaling pathway	2.36	2.02
Renal cell carcinoma	1.95	1.63
Ras signaling pathway	1.77	1.46
Progesterone-mediated oocyte maturation	1.72	1.43

HTLV-I infection	1.64	1.36

1.4 ADME

Table 8. SMILES used to calculate descriptors for the computational studies.

Name	SMILES
BAG	COC(=O)CNC(=O)CCNC(=O)OC(C)(C)C
BABA	COC(=O)C(C)NC(=O)CCNC(=O)OC(C)(C)C
BAV	COC(=O)C(NC(=O)CCNC(=O)OC(C)(C)C)C(C)C
BAL	COC(=O)C(CC(C)C)NC(=O)CCNC(=O)OC(C)(C)C
BAI	CCC(C)C(NC(=O)CCNC(=O)OC(C)(C)C)C(=O)OC
BAF	COC(=O)C(CC1=CC=CC=C1)NC(=O)CCNC(=O)OC(C)(C)C
BAE	COC(=O)CCC(NC(=O)CCNC(=O)OC(C)(C)C)C(=O)OC
BAD	COC(=O)CC(NC(=O)CCNC(=O)OC(C)(C)C)C(=O)OC
BAR	COC(=O)[C@H](CCCNC(=N)N[N+]([O-])=O)NC(=O)CCNC(=O)OC(C)(C)C
HG	COC(=O)CNC(=O)C(CC1=C[N](CC2=CC=CC=C2)C=N1)NC(=O)OC(C)(C)C
HA	COC(=O)C(C)NC(=O)C(CC1=C[N](CC2=CC=CC=C2)C=N1)NC(=O)OC(C)(C)C
HV	COC(=O)C(NC(=O)C(CC1=C[N](CC2=CC=CC=C2)C=N1)NC(=O)OC(C)(C)C)C(C)C
HL	COC(=O)C(CC(C)C)NC(=O)C(CC1=C[N](CC2=CC=CC=C2)C=N1)NC(=O)OC(C)(C)C
HI	CCC(C)C(NC(=O)C(CC1=C[N](CC2=CC=CC=C2)C=N1)NC(=O)OC(C)(C)C)C(=O)OC
HF	COC(=O)C(CC1=CC=CC=C1)NC(=O)C(CC2=C[N](CC3=CC=CC=C3)C=N2)NC(=O)OC(C)(C)C
HE	COC(=O)CCC(NC(=O)[C@H](CC1=C[N](CC2=CC=CC=C2)C=N1)NC(=O)OC(C)(C)C)C(=O)OC
HD	COC(=O)CC(NC(=O)[C@@H](CC1=C[N](CC2=CC=CC=C2)C=N1)NC(=O)OC(C)(C)C)C(=O)OC
HR	COC(=O)C(CCCNC(=N)N[N+]([O-<br>])=O)NC(=O)[C@@H](CC1=C[N](CC2=CC=CC=C2)C=N1)NC(=O)OC(C)(C)C
dHV	COC(=O)C(NC(=O)C([NH3+])CC1=C[NH]C=N1)C(C)C
dHR	COC(=O)C(CCCNC(=N)N[N+]([O-])=O)NC(=O)C([NH3+])CC1=C[NH]C=N1

Table 9. Predicted gastrointestinal absorption and blood-brain barrier penetration, BOILED-Egg graph-related numeric values.

Name	TPSA	WLogP	GIA	BBB
BAG	93.73	0.19	True	False
BAA	93.73	0.58	True	False
BAV	93.73	1.21	True	False
BAL	93.73	1.61	True	False
BAI	93.73	1.61	True	False
BAF	93.73	1.8	True	False
BAE	120.03	0.51	True	False
BAD	120.03	0.12	True	False
BAR	184.78	-0.36	False	False
HG	111.55	1.66	True	False
HA	111.55	2.04	True	False
HV	111.55	2.68	True	False

HL	111.55	3.07	True	False
HI	111.55	3.07	True	False
HF	111.55	3.27	True	False
HE	137.85	1.98	True	False
HD	137.85	1.59	True	False
HR	202.6	1.11	False	False
dHV	122.41	1.16	True	False
dHR	213.46	-0.41	False	False

Table 10 Prediction of TFAx dipeptidomimetics metabolic fate through ADME properties calculation.

Name	MW	H-Acceptors	H-Donors	LogP	TPSA	Heteroatoms	Rotatable Bonds	sp3 atoms	Heavy atoms	QED	Lipinski
**BAG**	260	5	2	0.190	93.73	7	5	0.727	18	0.688	True
**BAA**	274	5	2	0.579	93.73	7	5	0.750	19	0.715	True
**BAV**	302	5	2	1.215	93.73	7	6	0.786	21	0.719	True
**BAL**	316	5	2	1.605	93.73	7	7	0.800	22	0.696	True
**BAI**	316	5	2	1.605	93.73	7	7	0.800	22	0.696	True
**BAF**	350	5	2	1.802	93.73	7	7	0.500	25	0.732	True
**BAE**	346	7	2	0.512	120.03	9	8	0.733	24	0.485	True
**BAD**	332	7	2	0.122	120.03	9	7	0.714	23	0.502	True
**BAR**	404	8	5	-0.355	184.78	13	10	0.733	28	0.081	True
**HG**	416	7	2	1.656	111.55	9	8	0.429	30	0.633	True
**HA**	430	7	2	2.045	111.55	9	8	0.455	31	0.620	True
**HV**	458	7	2	2.681	111.55	9	9	0.500	33	0.559	True
**HL**	472	7	2	3.071	111.55	9	10	0.520	34	0.514	True
**HI**	472	7	2	3.071	111.55	9	10	0.520	34	0.514	True
**HF**	506	7	2	3.268	111.55	9	10	0.357	37	0.408	True
**HE**	502	9	2	1.978	137.85	11	11	0.480	36	0.351	True
**HD**	488	9	2	1.588	137.85	11	10	0.458	35	0.380	True
**HR**	560	10	5	1.111	202.6	15	13	0.480	40	0.059	True
**dHV**	269	4	3	-1.124	111.72	7	6	0.583	19	0.568	True
**dHR**	371	7	6	-2.694	202.77	13	10	0.538	26	0.064	True

2. SYNTHETIC STUDIES

Table 11. Synthesis efficacy shown in % up until the 3rd decimal place.

**1**	**Average** [%]
**BAG**	40.314
**BAA**	48.506
**BAV**	46.479
**BAL**	47.500
**BAI**	47.837
**BAF**	37.115
**BAE**	50.254
**BAD**	42.803
**HG**	45.191
**HA**	48.378
**HV**	73.466
**HL**	47.589
**HI**	47.927
**HF**	37.171
**HE**	50.368
**HD**	42.792
**HR**	45.191

4. Biological studies

Table 12. Initial MTT screening results for dipeptidomimetics able to kill more than 25% MCF7 breast cancer cells (10 out of 18 proposed), within the concentration range of 10 to 5000 μM.

CONCENTRATION	BAG	BAA	BAV	BAI	BAE	BAD	BAR	HA	HV	HR
**10**	68.2649	84.7987	72.34873	74.80056	80.2874	70.56864	72.21133	70.60833	77.5852	81.6156
**100**	84.53153	88.42453	80.8599	83.41707	68.7	67.17333	73.21893	82.9896	76.9669	94.4396
**500**	69.3412	58.395	58.9904	59.082	59.6316	66.3642	70.2572	67.0512	30.71653	65.0818
**1000**	67.0741	32.3119	55.876	64.99936	65.8146	52.8532	62.4941	61.372	7.6257	25.0984
**2500**	55.2348	18.0681	50.53267	25.67853	40.075	43.38787	22.3962	19.38867	2.09535	2.61976
**5000**	35.6324	0.60456	22.84504	33.2737	37.52983	16.4193	17.89635	8.46384	0.8244	0.29312

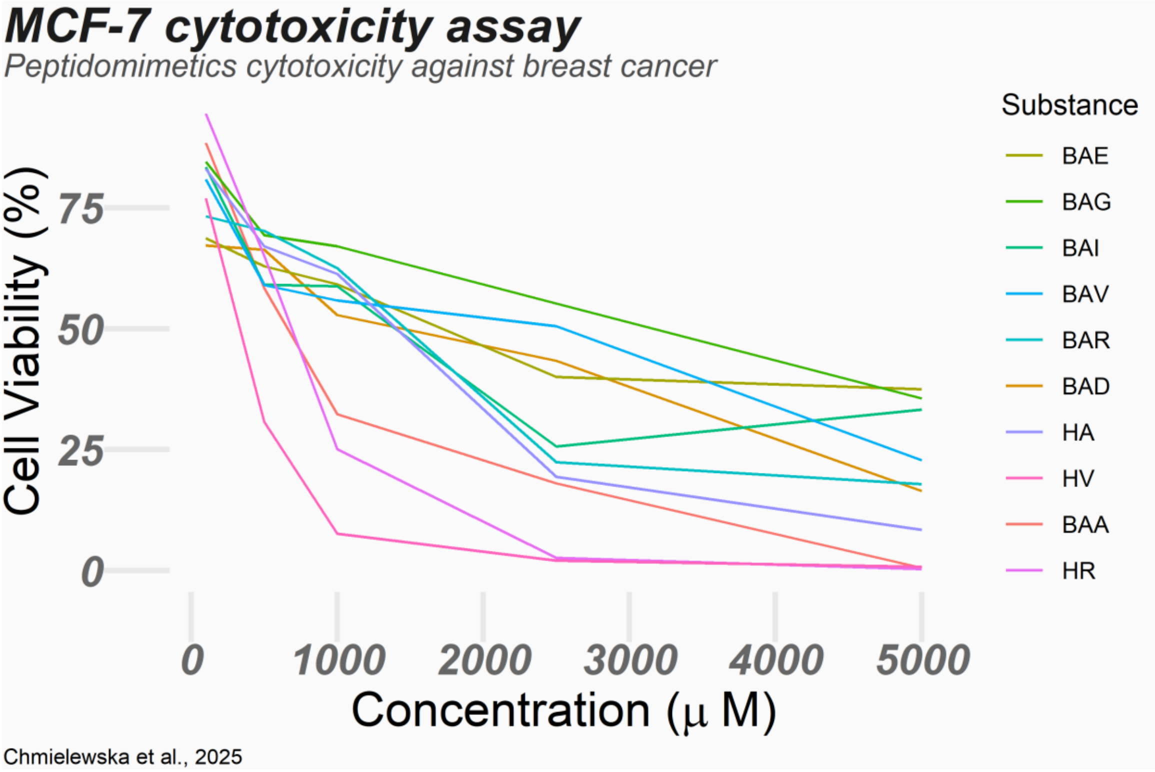

Fig 1. Cytotoxicity MTT *in vitro* test results (from Table 10) presenting a broader concentration range for 10 proposed dipeptidomimetics. Legend is sorted based on descending Viability acquired at 5000µM concentration.

Table 13. MTT cytotoxicity test (% of viable cells for 1mM concentration), n=3 from 3 independent experiments.

Name	MCF-7	HDFa
**BAA**	31.78	70.39
**HA**	95.12	95.83
**HV**	16.46	70.2
**BAF**	100	100
**HF**	76.06	78.48
**HR**	51.71	80.39
**Carnosine**	94.23	59.14
**dHV**	47.37	54.31
**dHR**	82.48	70.68

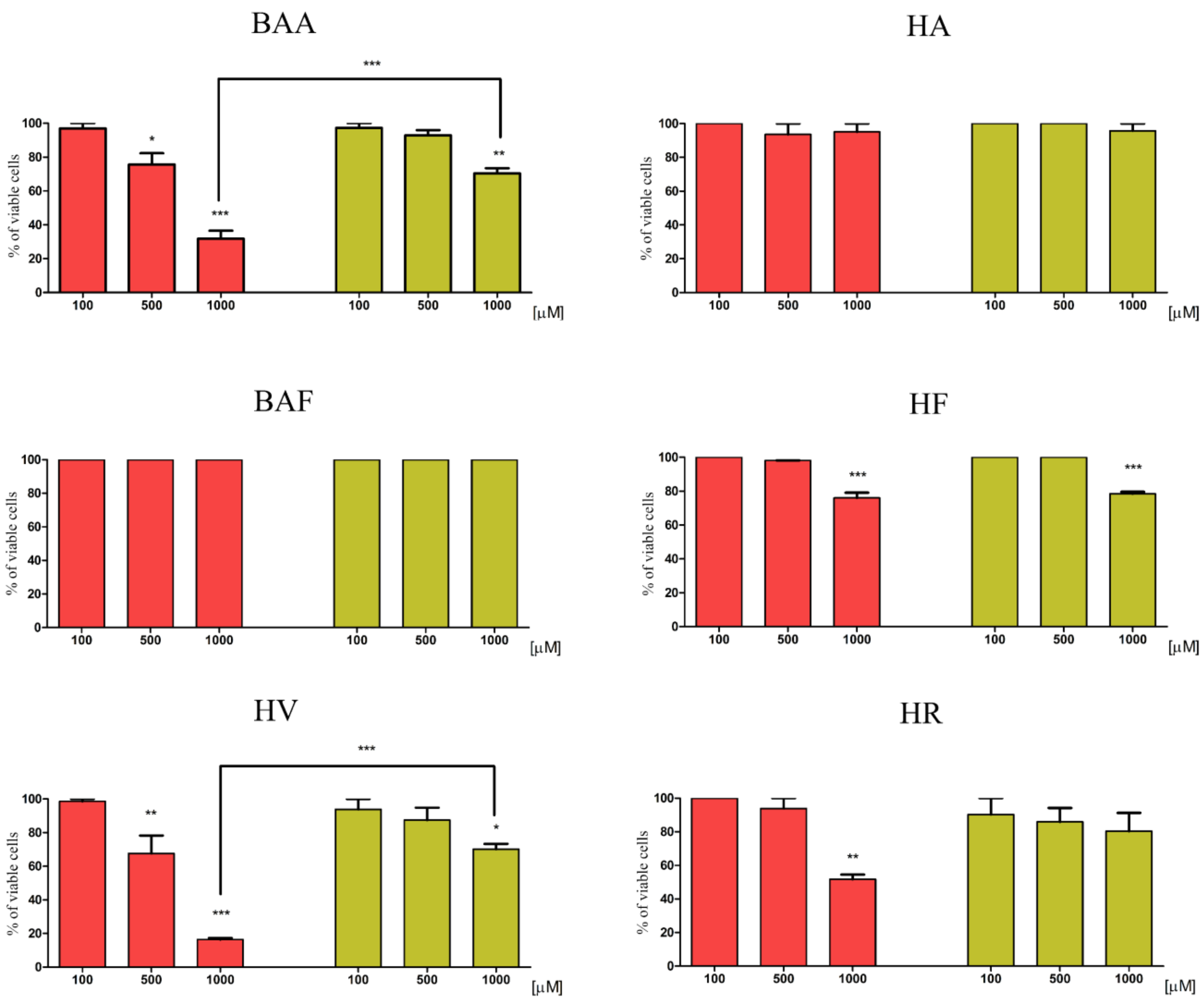

Fig 2. Cytotoxicity MTT *in vitro* test results (expanded Table 12) presenting cytotoxicity of 6 proposed dipeptidomimetics against MCF7 and HDFa cell lines.
